# Comparative Genomic Analyses Provide New Insights into the Evolutionary Dynamics of Heterochromatin in *Drosophila*

**DOI:** 10.1371/journal.pgen.1006212

**Published:** 2016-08-11

**Authors:** Ruggiero Caizzi, Roberta Moschetti, Lucia Piacentini, Laura Fanti, Renè Massimiliano Marsano, Patrizio Dimitri

**Affiliations:** 1 Dipartimento di Biologia, Università degli Studi di Bari, Bari, Italy; 2 Istituto Pasteur Italia-Fondazione Cenci Bolognetti, Dipartimento di Biologia e Biotecnologie ‘‘Charles Darwin”, Sapienza Università di Roma, Roma, Italy; Lawrence Berkeley National Lab/UC Berkeley, UNITED STATES

## Abstract

The term heterochromatin has been long considered synonymous with gene silencing, but it is now clear that the presence of transcribed genes embedded in pericentromeric heterochromatin is a conserved feature in the evolution of eukaryotic genomes. Several studies have addressed the epigenetic changes that enable the expression of genes in pericentric heterochromatin, yet little is known about the evolutionary processes through which this has occurred. By combining genome annotation analysis and high-resolution cytology, we have identified and mapped 53 orthologs of *D*. *melanogaster* heterochromatic genes in the genomes of two evolutionarily distant species, *D*. *pseudoobscura* and *D*. *virilis*. Our results show that the orthologs of the *D*. *melanogaster* heterochromatic genes are clustered at three main genomic regions in *D*. *virilis* and *D*. *pseudoobscura*. In *D*. *virilis*, the clusters lie in the middle of euchromatin, while those in *D*. *pseudoobscura* are located in the proximal portion of the chromosome arms. Some orthologs map to the corresponding Muller C element in *D*. *pseudoobscura* and *D*. *virilis*, while others localize on the Muller B element, suggesting that chromosomal rearrangements that have been instrumental in the fusion of two separate elements involved the progenitors of genes currently located in *D*. *melanogaster* heterochromatin. These results demonstrate an evolutionary repositioning of gene clusters from ancestral locations in euchromatin to the pericentromeric heterochromatin of descendent *D*. *melanogaster* chromosomes. Remarkably, in both *D*. *virilis* and *D*. *pseudoobscura* the gene clusters show a conserved association with the HP1a protein, one of the most highly evolutionarily conserved epigenetic marks. In light of these results, we suggest a new scenario whereby ancestral HP1-like proteins (and possibly other epigenetic marks) may have contributed to the evolutionary repositioning of gene clusters into heterochromatin.

## Author Summary

This study concerns the evolutionary dynamics underlying the emergence of heterochromatic single-copy genes in *D*. *melanogaster* heterochromatin. By combining genome annotation analysis and high-resolution cytology, we have performed a comparative mapping of the orthologs of 53 single-copy genes of *D*. *melanogaster* heterochromatin, in the genomes of *D*. *pseudoobscura* and *D*. *virilis* evolutionarily distant species. The results of our work are consistent with a scenario where the *D*. *melanogaster* heterochromatin genes arose through an evolutionary repositioning from a euchromatic location (*D*. *virilis*) to heterochromatin, passing through an intermediate location in regions associated with distal heterochromatin (*D*. *pseudoobscura*). The previously unanticipated observation, that in both *D*. *virilis* and *D*. *pseudoobscura* the gene clusters show a striking association with the HP1a protein, is remarkable in that it implies that the HP1 epigenetic mark may have contributed to the the success of repositioning of genes to pericentromeric heterochromatin by protecting them against silencing effects.

## Introduction

The organization of eukaryotic genomes into euchromatin and heterochromatin represents one of the most important and still unsolved aspects of genome evolution. Heterochromatin was originally identified from the early observation that specific regions of interphase nuclei possess distinctive staining properties [1]; it remains an elusive component of the eukaryotic genome. Our understanding of its nature and properties has progressively expanded through decades of intensive research, initially in cytological and classical genetic studies and later by sophisticated molecular and *in silico* techniques. Key steps have been the identification of two types of heterochromatin, constitutive and facultative [2]; the discovery of the unique genetic properties of heterochromatin [3,4,5,6,7]; the mapping of satellite DNAs, transposable elements and other repeated sequences in the heterochromatin [8,9,10,11,12]; the recent advances in whole genome sequencing [13,14,15]; and the systematic analysis of heterochromatin-binding proteins [16,17], and of histone modifications in heterochromatin [18,19].

However, our knowledge of heterochromatin is far from being exhaustive or satisfactory. For example, dozens of essential genes and hundreds of putative genes have been identified in the heterochromatin of *D*. *melanogaster* [12,13,14,15], but their existence is quite paradoxical [6,7]. In fact, a signature feature of heterochromatin is its ability to silence euchromatic genes that are brought within a heterochromatic environment following a chromosome rearrangement or a transposition event, a well-known phenomenon called position effect variegation (PEV) which provides an important model for studying the mechanisms regulating gene repression by chromatin modifications [20,21,22,23,24].Yet, the single-copy genes embedded in the heterochromatin of *D*. *melanogaster* are bound by specific proteins [25,26,27], show a pattern of modified histones [18] and their proper expression depends on their heterochromatic location [28,29], despite the fact that they are transcribed from promoter regions sharing basic similarities with those of euchromatic genes [30,31].

To our knowledge, the evolutionary history of the *D*. *melanogaster* heterochromatic genes has only been addressed in two studies, that have investigated the chromosomal location in *D*. *pseudoobscura* and *D*. *virilis* of: i) a small cluster of genes, including the *light* gene, located in the heterochromatin of chromosome 2 [30], and ii) the two adjacent *RPL15* and *Dbp80* genes located in the heterochromatin of chromosome 3 [31]. In both cases, the counterparts of *D*. *melanogaster* genes were shown to map in euchromatic regions in *D*. *pseudoobscura* and in *D*. *virilis*, suggesting a repositioning of these genes during genome evolution in the Drosophilidae lineage.

Comparative studies of genes located on the dot chromosomes of *D*. *melanogaster and D*. *virilis*, both of which show heterochromatic properties, have shown that most of the genes maintain an overall synteny and only rare instances of “wanderer” genes (present in a euchromatic chromosome arm in one species and on the dot chromosome in the other) were found [32]. However, although the *D*. *melanogaster* dot chromosome 4 is considered heterochromatic, it shares only certain properties with the pericentric regions of the large autosomes, and genes located there are likely to be under different gene expression constraints [33].

**Fig 1:**
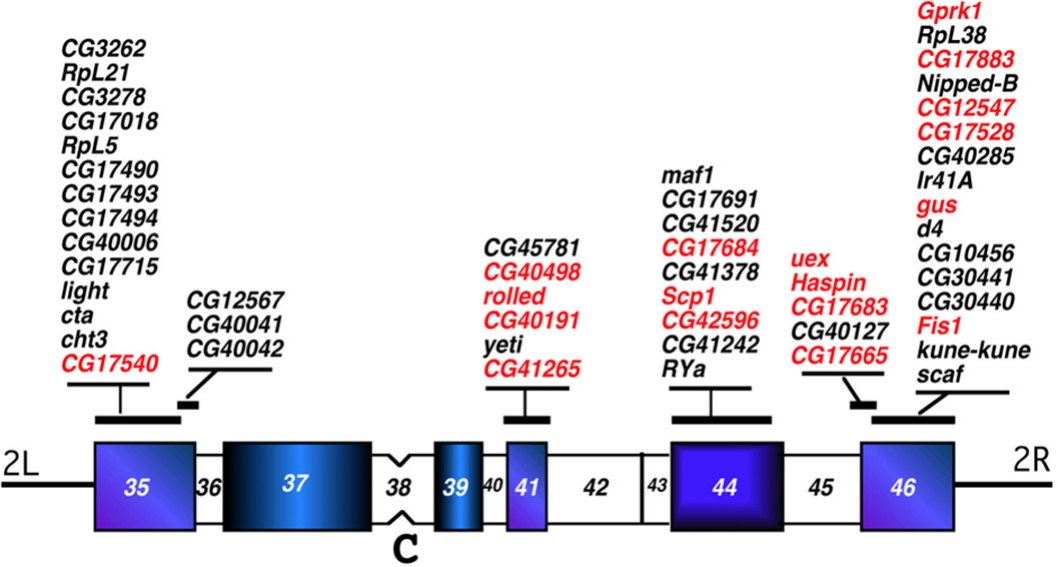
Cytogenetic map of the heterochromatin of chromosome 2 in *D*. *melanogaster*. In mitotic chromosome preparations of *D*. *melanogaster*, the pericentric heterocromatin has been differentiated into cytogically defined bands using specific chromosome banding techniques [3]. The heterochromatic regions of the left arm of chromosome 2 (2Lh) are numbered from h35 to h37, whereas those of the right arm of chromosome 2 (2Rh) are numbered from h39 to h46. Region h38 indicates the centromere (C). While most heterochromatin genes of the 2L arm appears to be restricted to region h35 (polytenic region 40), genes from the right arm are more scattered being localized in h41, h44 and h46 [52] and thus are separated by megabases of satellite DNA blocks and other repeated DNA sequences [12,13,15]. Only the mapping of the heterochromatic genes studied in this work is reported (see Table 1). For a more detailed map see Dimitri et al. [52]. The mapping of a group of Drosophila heterochromatin gene orthologs in *D*. *virilis* and *D*. *pseudoobscura* was retrieved from the work of Schaeffer et al, (supplemental Tables 21 and 24, [35]; these genes are shown in black, while the orthologs mapped in this work are shown in red. The heterochromatin of chromosome 2Rh was estimated to contain at least 12.1 Mb DNA [14], while according to the genomic coordinates of Release 6.03 it encompasses 5.2 Mb. This apparent discrepancy can be explained considering that heterochromatin scaffolds (named 2R-Het or 2R-U in the previous Release 5.1 [48] have been incorporated as an uninterrupted sequence in Release 6.03, without taking into account that those scaffolds are separated by megabases of satellite DNA stretches that are not included in the sequence [12, 13, 15].

To investigate the evolutionary dynamics underlying the emergence of heterochromatic genes in *D*. *melanogaster*, we identified and mapped their orthologs in *D*. *pseudoobscura* and *D*. *virilis* genomes. We took advantage of the extensive whole genome sequencing data currently available for at least 12 Drosophilidae species and of recent comparative studies confirming the overall conservation of chromosomal synteny in Drosophilidae [34,35,36]. In particular, we focused our analyses on 53 single-copy genes mapped to the heterochromatin of chromosome 2 of *D*. *melanogaster* (Fig 1). This genomic region corresponds to about 18.3 Mb and has been extensively characterized in the last decades at genetic, cytological and molecular levels for the presence of essential and putative genes and other genetic loci [37,38,39,40,41].

Our comparative studies show that the orthologs of chromosome 2 heterochromatic genes of *D*. *melanogaster* tend to be clustered at specific regions of separate chromosomal elements of *D*. *pseudoobscura* and *D*. *virili*s. These results suggest that the repositioning of genes to pericentric heterochromatin and their interspersion with numerous heterochromatic sequences, as seen in *D*. *melanogaster*, may have originated concomitantly with the rearrangements leading to the centric fusion of two separate chromosomal elements during the evolution of the Drosophilidae karyotype [35,42,43,44]. We also have data suggesting that ancestral HP1-like proteins may have contributed to the evolutionary repositioning of gene clusters to pericentromeric heterochromatin.

## Results

### Searching for chromosome 2 heterochromatic gene orthologs in *Drosophila* species

**Fig 2:**
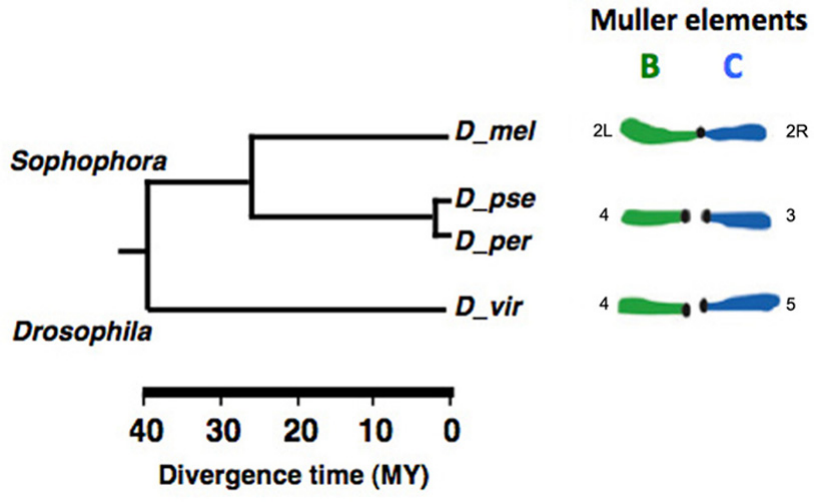
Phylogenetic relationships among syntenic chromosomes of the analysed *Drosophila* species. The scheme shows the phylogenetic relationships between *D*. *melanogaster*, *D*. *pseudoobscura*, its sibling species *D*. *persimilis* and *D*. *virilis*, as defined by Muller [45]. The syntenic Muller elements (right) are shown by the number of their corresponding chromosomes.

The phylogenetic relationships among the syntenic chromosomes as defined by Muller in *D*. *melanogaster*, *D*. *pseudoobscura*, its sibling species *D*. *persimilis* and *D*. *virilis* [45] are shown in Fig 2. These species share a common ancestor dated around 40–50 million years ago, their genomic assembly is almost complete, and thus they are very useful for comparative genomic studies. Since *D*. *pseudoobscura* and *D*. *persimilis* genomes show a very low level of divergence [46], *D*. *persimilis* was included in this study as a useful source of information to compensate for the absence or partial lack of data from *D*. *pseudoobscura*.

The cytogenetic location of *D*. *melanogaster* heterochromatic genes studied in this paper is shown in the mitotic heterochromatin map of chromosome 2 (Fig 1, Table 1) [3]. The most distal mitotic bands, h35 (2L, left arm) and h46 (2R, right arm), are the only heterochromatic regions that can be resolved on Bridges’ polytene map and contain the transition zone between heterochromatin and euchromatin [39,40,47]. The euchromatin-heterochromatin cytogenomic borders, as defined by Hoskins et al [13,14] and by Riddle et al [19], fall within these regions, and all the genes reported in Fig 1 should be considered embedded in the pericentromeric heterochromatin of chromosome 2, with the exception of CG1298 and CG11066 that are at 40 Kb outside the 2Rh cytogenomic border. The genomic coordinates of regions containing the examined genes are: from CG3262 to CG40042 (2Lh): 22,239,906..23,313,578; from CG45781 to CG11066 (2Rh): 432,219..5,691,723.

**Table 1:** Mapping of the analysed *D*. *melanogaster* heterochromatic genes and their orthologs in *D*. *pseudoobscura* and *D*. *virilis*.

Gene name in D.mel	Cyto map in D.mel	in D.pse	In D.vir
CG3262	**2L h35, 40F6**	**83A**	**47C**
CG12775 (*RpL21*)	**2L h35, 40F6**	**83A**	**47C**
CG3278 (*Tif-IA*)	**2L h35, 40F6-7**	**83A**	**47C**
CG17018	**2L h35, 40F7**	**83A**	**47C**
CG17489 (*RpL5*)	**2L h35, 40F7**	**83A**	**47C**
CG17490	**2L h35, 40F7**	**83A**	**47C**
CG17493	**2L h35, 40F7**	**96B**	**47C**
CG17494	**2L h35, 40F7**	**83A**	**47C**
CG40006	**2L h35**	**83A**	**47C**
CG17715	**2L h35**	**89B**	**47C**
CG18028 (*light*)	**2L h35**	**83A**	**47C**
CG17678 (*cta*)	**2L h35**	**83A**	**49A**
CG18140 (*cht3*)	**2L h35**	**89B**	**47C**
CG17540 (*spf45*)	**2L h35**	83A*	**47C**
CG12567	**2L h35**	**83A**	**47C**
CG40041 (*gpb5*)	**2L h35**	**83A**	**47C**
CG40042	**2L h35**	**83A**	**47C**
**CENTROMERE**			
CG45781	**2R**	82AB*	**42F**
CG40498	**2R h41**	82AB*	**42F**
CG12559 (*rolled*)	**2R h41**	82AB*	**42F**
CG40191	**2R h41**	89B*	47C*
CG40218 (*Yeti*)	**2R h41**	**63A**	**53D**
CG41265 (*l(2)_41AB*)	**2R h41**	94A*	42F*
CG40196 (*maf1*)	**2R h44**	NA	**42F**
CG17691	**2R h44**	82AB*	**42F**
CG41520	**2R h44**	NA	**55D**
CG17684	**2R h44**	93D*	**42F**
CG41378	**2R h44**	NA	**43A**
CG15848 (*Scp1*)	**2R h44**	82AB*	**42F**
CG42596	**2R h44**	93A*	42F*
CG41242	**2R h44**	63A*	**55D**
CG40733 (*RYa*)	**2R h44**	**63A**	**55D**
CG42595 (*uex*)	**2R h46**	82AB*	43A*
CG40080 (*Haspin*)	**2R h46**	82AB*	**43A**
CG17683	**2R h46**	83A*	47C*
CG40127	**2R h46**	**63A**	**55D**
CG17665 (*Int3*)	**2R h46**	82AB*	43A*
CG40129 (*Gprk1*)	**2R h46, 41B3-C2**	63A*	**59F**
CG18001 (*RpL38*)	**2R h46, 41A2**	NA	**55D**
CG17883	**2R h46, 41B3**	82AB*	**42F**
CG17704 (*Nipped-B*)	**2R h46, 41B3-C1**	NA	**43A**
CG12547	**2R h46, 41C2**	83A*	**43A**
CG17528	**2R h46, 41C2**	83A*	47C*
CG40285	**2R h46, 41C3**	**63A**	**42C**
CG33492 (*Ir41A*)	**2R h46, 41C6-D1**	**63A**	**55D**
CG2944 (*gus*)	**2R h46, 41D13**	82AB*	**42F**
CG2682 (*d4*)	**2R h46, 41E3-4**	**63A**	**55D**
CG10465	**2R h46, 41E5**	**63A**	**55D**
CG30441	**2R h46, 41E5**	**63A**	**55D**
CG30440	**2R h46, 41F2**	**63A**	**55D**
CG17510 (*Fis1*)	**2R h46, 41F5**	82AB*	**43A**
CG1298 (kune-kune)	**2R h46, 41F6**	**63A**	**55D**
CG11066 (*scaf*)	**2R h46, 41F6-8**	**63A**	**53D**

* = mapped by FISH in this study; no asterisk = retrieved from the dataset of Schaeffer et al [35]

NA, Not Assigned

Heterochromatic genes, as well as numerous euchromatic genes, have been annotated as having orthologs in several *Drosophila* species [36,48]. A database, Ortho DB, is easily accessible and represents an important source of information [49]. However, at present a large number of orthologs of *D*. *melanogaster* heterochromatic genes have been only partially annotated or not yet identified in other *Drosophila* species.

Using tBLASTn (see Materials and Methods), we were able to retrieve ortholog candidates of 9 *D*. *melanogaster* heterochromatic genes in *D*. *pseudoobscura*, *D*. *persimilis* and *D*. *virilis*. Some were already annotated, while others were not. All 9 retrieved genes appear to be true orthologs based on the following features: 1) strong sequence similarities in coding regions and exon-intron structure among the analyzed species; 2) high level of identity of deduced proteins; 3) no evidence of related duplicated sequences within the genomes found by FISH or BLAST analyses.

The identified *D*. *melanogaster* heterochromatic gene orthologs with their genomic coordinates are listed in Table 2 and structural comparisons between species are shown in S1 Fig.

**Table 2:** List of *D*. *melanogaster* 2R-HET genes analysed in *D*. *pseudoobscura*, *D*. *persimilis* and *D*. *virili*s.

D. melanogaster	D. pseudoobscura scaffold position (annotated gene)	D. persimilis scaffold position (annotated gene)	D. virilis scaffold position (annotated gene)
CG40498 (2R, h41)	Unknown_gr_17: CH475486 1,840..2,954 (-)	sc_16: CH479195 162,581..163,648 (-)	sc_12963: CH940649 14,007,329..14,009,185 (-)
CG12559 (2R, h41) *(rolled)*	U_gr_48: 16,184..16,341 (-) (exon 1) U_gr_246: 5,480..5,783 (+) (exon 2,3)	sc_89: 38,424..82,946 (-) (**GL20446)**	sc_12963: 14,128,775..14,132,351 (+) (GJ17518)
CG41265 (2R, h41) *(l(2)41Ab)*	4_gr_3: 1,542..2,613 (+) (exons 5,6)	sc_1: CH479180 1,480,916..1,485,248 (+)	sc_12963: 14,140,113..14,143,403 (-) (GJ21982)
CG15848 (2R, h44) *(Scp1)*	U_singl_2543: 392..442 (-) (exon 1) U_gr_418: 3,739..3,816 (+) (exon 3) U_gr_462: 5,833..6,167 (-) (exons 4,5)	sc_70: 271,588..290,093 (+) (**GL24797)**	sc_12963: CH940649 14,279,373..14,281,827 (+)
CG42595 (2R, h44-46) *(uex*, *l(2)41Ad)*	Not found	sc_16: CH479195 262,368..267,520 (+)	sc_12963: 13,968..13,979,103 (-) (GJ22088)
CG40080 (2R, h44-46) *(Haspin)*	Unknown_gr_17: 10,931..12,994 (-) (GA25640)	sc_16: 174,905..176,472 (-) (**GL21152)**	sc_12963: 14,010,005..14,012,197 (-) (GJ22035)
CG17665 (2R, h44-46) *(IntS3)*	Unknown_gr_493: 5,288..9,439 (+)	sc_16: 246,115..253,680 (+) (**GL21164)**	sc_12963: 13,990,844..13,995,254 (+) (GJ17515))
CG12547 (2R, h46)	Not found	sc_16: 250,917..256,140 (-) **(GL21149)**	sc_12963: 13,979,607..13,982,358 (+) (GJ17513)
CG2944 (2R, h46) *(gus)*	Unknown_gr_200: 14,240..19,049 (+) (GA23998)	sc_125: 75,000..78,723 (+) (**GL15433)**	sc_12963: 14,257,527..14,265,541 (-) (GJ21895)

### Mapping *D*. *melanogaster* heterochromatic gene orthologs in *D*. *pseudoobscura* and *D*. *virilis*

Schaeffer et al. [35] produced an integrated physical and cytogenetic map of 11 species of the *Drosophila* genus, anchoring the genome assembly scaffolds to the polytene chromosome map.

We first analyzed in detail the genomic coordinates of the assembled scaffolds containing specific marker genes [Tables S21 and S24 in ref. 35], to retrieve the polytene chromosome maps of a group of *D*. *melanogaster* heterochromatic gene orthologs in both *D*. *pseudoobscura* and *D*. *virilis* (Table 1). To confirm these data and to extend mapping to a larger number of orthologs, we performed fluorescent *in situ* hybridization (FISH) on polytene chromosomes.

**Fig 3:**
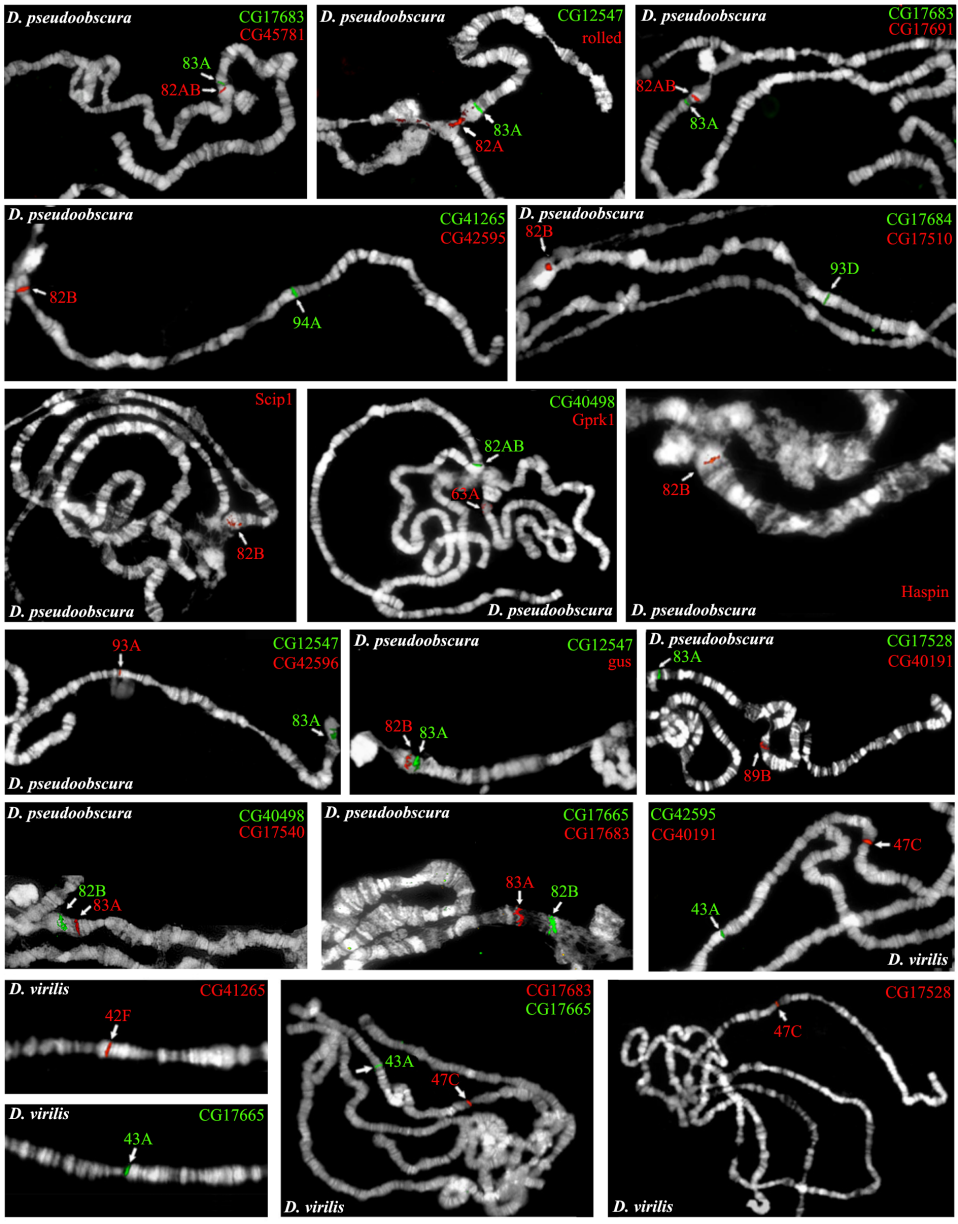
Examples of FISH mapping of *D*. *melanogaster* heterochromatin gene orthologs. The panels show single or double-colour FISH mapping of orthologs to polytene chromosomes of *D*. *pseudoobscura* and *D*. *virilis* using specific PCR probes. Each ortholog is designated with the name of the corresponding *D*. *melanogaster* gene.

For each gene, we cloned PCR fragments to be used as specific probes (see Mat and Met). Examples of FISH mapping are shown in Fig 3 and the results are summarized in Table 1.

**Fig 4:**
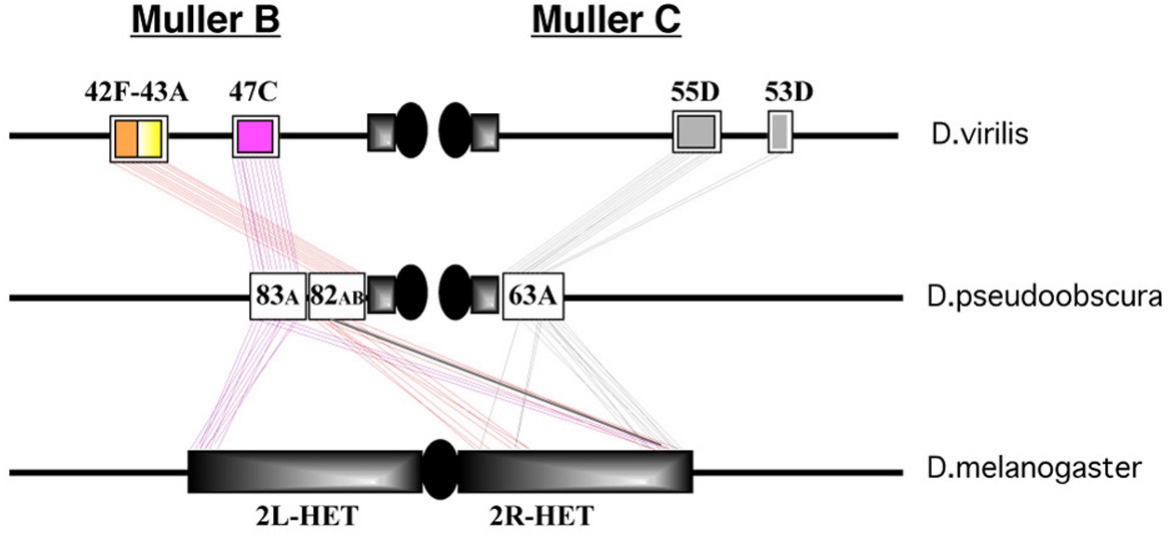
Schematic representation showing the syntenic chromosomal regions of *D*. *virilis* and *D*. *pseudoobscura* containing the *D*. *melanogaster* heterochromatic gene orthologs. Black boxes correspond to heterochromatin regions; black oval shape indicates the centromere.

It is clear that the *D*. *melanogaster* heterochromatic orthologs tend to be clustered at specific regions of the *D*. *pseudoobscura* and *D*. *virilis* genomes (Table 2 and Fig 4). In *D*. *virilis* three main clusters were found at polytene regions 47C, 42F-43A and 55D, that we will call Dvir_47C, Dvir_42F-43A and Dvir_55D, respectively. Dvir_47C and Dvir_42F-43A are on chromosome 4 (Muller B element), while Dvir_55D is on chromosome 5 (Muller C element). The Dvir_47C harbors 16 orthologs of *D*. *melanogaster* heterochromatin genes located in 2Lh, together with at least 3 other orthologs from 2Rh. Dvir_42F-43A contains 18 orthologs from 2Rh, while Dvir_55D harbors 11 orthologs from 2Rh (mainly localized in region h46 of the mitotic map).

In *D*. *pseudoobscura* three main clusters were also found at polytene regions 63A, 83A and 82AB, that we will call Dpse_63A, Dpse_83A and Dpse_82AB. The Dpse_83A and Dpse_82AB main clusters on the Muller B element share at least 15 and 11 orthologs with the Dvir_47C and Dvir_42F-43A clusters on Muller B, respectively. The Dpse_63A Muller C shares 9 orthologs with the Dvir_55D main cluster and 2 with the Dvir_53D minor site on the Muller C element.

In addition, 12 *D*. *melanogaster* heterochromatic gene orthologs were not found in the above mentioned clusters and map to different locations in both the *D*. *virilis* (CG40285 at Dvir_42C; CG40129 at Dvir_59F; CG17678 at Dvir_49A; CG40218 and CG11066 at Dvir_53D) and *D*. *pseudoobscura* (CG17493 at Dpse_96B; CG41265 at Dpse_94A; CG17684 at Dpse_93D; CG42596 at Dpse_93A; CG17715, CG18140 and CG40191 at Dpse_89B). With the exception of the CG17715, CG18140 and CG40191 orthologs, found together at Dpse_89B, and CG40218 and CG11066 found together at Dvir_53D, the other 7 orthologs represent evidence of individual gene movements.

Our analysis of *D*. *melanogaster* heterochromatic gene orthologs in *D*. *virilis* and *D*. *pseudoobscura* shows that i) a cluster of orthologs retained its location on Muller B and Muller C elements in the three species analyzed; ii) a group of 14 *D*. *melanogaster* Muller C heterochromatic genes were originally located in the Muller B element (Table 1 and Fig 4) of *D*. *virilis* and *D*. *pseudoobscura*

### Analysis of syntenic relationships among *Drosophila* species

**Fig 5:**
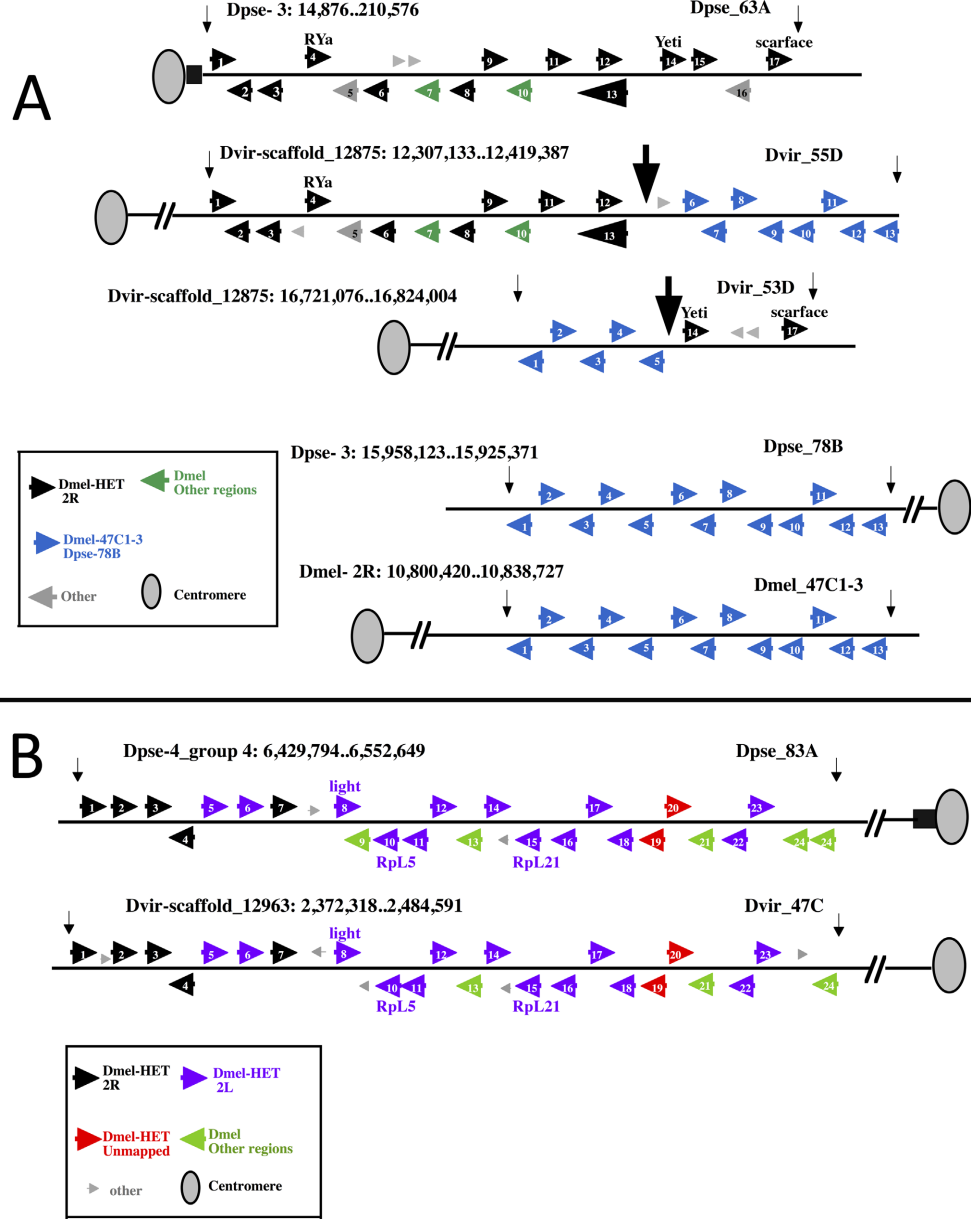
Syntenic relationships of gene clusters. (A) Syntenic relationships between Dpse_63A, Dvir_55D and Dvir_53D regions. Genes are depicted as arrowheads showing the transcription orientation and coloured according to their localization in *D*. *melanogaster* (box). Small arrows delimit the analyzed scaffold regions. The *D*. *melanogaster* gene name is shown for three genes used as markers (*RYa*, *Yeti* and *scarface*). Big arrows at the Dvir_55D and Dvir_53D in *D*. *virilis* chromosomes show the breakpoints of to the hypothetical recombination sites leading to the gene arrangement at the region Dpse_63A and Dpse_78B in *D*. *pseudoobscura*. The Dpse_78B cluster is syntenic with the Dmel_47C1-3 in *D*. *melanogaster* (lower row). The list of the genes numbered in the arrowheads is reported in Table 3 and Table 4. (B) Syntenic relationships between Dpse_83A and Dmel_47C regions. Genes are depicted as arrowheads showing the transcription orientation and coloured according to their localization in *D*. *melanogaster* (box). Small arrows delimit the scaffold region analized. The *D*. *melanogaster* gene name is shown for three genes used as markers (*light*, *RpL5* and *RpL21*). The complete list of genes numbered in the arrowheads is shown in Table 5.

To explore in more detail the gene content and syntenic relationships between the clusters identified by FISH, we compared annotated genes of *D*. *pseudoobscura* with their orthologs in *D*. *virilis* and *D*. *melanogaster*, looking at the relative direction of transcription. The genes we examined are shown in Tables 3, 4 and 5, where they are listed using the species-specific designation. Each gene was also colored coded on the basis of its position in the genome of *D*. *melanogaster*. The results of the analysis are depicted in Fig 5.

**Table 3:** List of *D*. *pseudoobscura* genes at Dpse_63A orthologous to genes of *D*. *virilis* and *D*. *melanogaster*.

	D_mel		Dpse_63A	D_vir	
	Name	Chr. position		Name	Chr. position
**1**	CG10395	2R-HET (41E5)	**GA10297**	GJ21913	55D
**2**	CG30441	2R-HET (41E5)	**GA15848**	GJ20418	55D
**3**	CG10465	2R-HET (41E5)	**GA10330**	GJ20417	55D
**4**	CG40733 (*RYa*)	2R-HET	**GA24590**	GJ21914	55D
**5**	-	-	**GA32996**	GJ20414	55D
**6**	CG3107	2R-HET (41D3)	**GA15984**	GJ20413	55D
	-	-	**GA31014**	-	-
	-	-	**GA31396**	-	-
**7**	CG33262	3L, 70A1	**GA25598**	GJ20411	55D
**8**	CG2682	2R-HET (41E4)	**GA15428**	GJ20409	55D
**9**	CG1298	2R-HET (41F6)	**GA11952**	GJ21915	55D
**10**	CG3781*	X, (5E3)	**GA24587**	GJ20408	55D
**11**	CG40127	2R-HET	**GA24591**	GJ21916	55D
**12**	CG33492 (*Ir41A*)	2R-HET (41C6)	**GA24592**	GJ21918	55D
**13**	CG30440	2R-HET (41F2)	**GA15847**	GJ20406	55D
**14**	CG40218 (*Yeti*)	2R-HET	**GA24593**	GJ22228	53D
**15**	CG30438	2R-HET (41F2)	**GA24594**	-	-
**16**	-	-	**GA24586**	-	-
**17**	CG11066 (*scarface*)	2R-HET (41F6)	**GA10737**	GJ22229	53D

Both mitotic and polytene chromosome map of *D*. *melanogaster* heterochromatic genes are indicated.

The gene marked by the asterisk is intronless

**Table 4:** List of *D*. *pseudoobscura* genes at Dpse_78B orthologous to genes of *D*. *virilis* and *D*. *melanogaster*.

	D_mel		Dpse_78B	D_vir	
	Name	Chr. position		Name	Chr. position
**1**	CG12340	2R, 47C1	**GA25025**	GJ20126	53D
**2**	CG12935	2R, 47C1	**GA11921**	GJ22225	53D
**3**	CG7637	2R, 47C1	**GA20497**	GJ20125	53D
**4**	CG12936	2R, 47C1	**GA11922**	GJ22227	53D
**5**	CG12341	2R, 47C1	**GA11566**	GJ20124	53D
**6**	CG7222	2R, 47C1	**GA20191**	GJ21920	55D
**7**	CG12342	2R, 47C1	**GA11567**	GJ20405	55D
**8**	CG12323	2R, 47C1	**GA11556**	GJ21921	55D
**9**	CG12938	2R, 47C1	**GA11923**	GJ20404	55D
**10**	CG12343	2R, 47C1	**GA11568**	GJ20403	55D
**11**	CG12325	2R, 47C1	**GA11557**	GJ21922	55D
**12**	CG12344	2R, 47C1	**GA11569**	GJ20402	55D
**13**	CG7686	2R, 47C3	**GA25022**	GJ20401	55D

**Table 5:** List of *D*. *pseudoobscura* genes at Dpse_83A orthologous to genes of *D*. *virilis* and *D*. *melanogaster*.

	D_mel		Dpse_83A	D_vir	
	Name	Chr. position		Name	Chr. position
**1**	CG17683 (*l(2)41Ae*)	2R-HET	**GA29080**	GJ13047	47C
**2**	CG8245	2R-HET (41F6)	**GA20925**	GJ13069	47C
**3**	CG8426	2R-HET (41F6)	**GA21070**	GJ13081	47C
**4**	CG1344	2R-HET (41F6)	**GA28759**	GJ15077	47C
**5**	CG17540	2L-HET	**GA29081**	GJ13092	47C
**6**	CG17494	2L-HET (40F7)	**GA29082**	GJ13013	47C
**7**	CG17528	2R-HET (41C2)	**GA29083**	GJ13114	47C
		-	**GA29084**	-	-
**8**	CG18028 (*light*)	2L-HET	**GA29085**	GJ13125	47C
**9**	CG8734	2R, (44D5)	**GA28758**	-	47C
**10**	CG17489 (*RpL5*)	2L-HET (40F7)	**GA28757**	GJ15046	47C
**11**	CG17490	2L-HET (40F7)	**GA30044**	GJ26463	47C
**12**	CG3262	2L-HET (40F6)	**GA17025**	GJ13135	47C
**13**	CG1142	3R, (84D8)	**GA28756**	GJ15035	47C
**14**	CG3278	2L-HET (40F6)	**GA17133**	GJ13145	47C
	-	-	**GA30035**	GJ15024	47C
**15**	CG12775 (*RpL21*)	2L-HET (40F6)	**GA11806**	GJ15013	47C
**16**	CG12567	2L-HET	**GA30010**	GJ15002	47C
**17**	CG40042	2L-HET	**GA29086**	GJ13156	47C
**18**	CG40041	2L-HET	**GA30008**	GJ14991	47C
**19**	CG12423	Unmapped Scaffold_8	**GA30009**	GJ14979	47C
**20**	CG17878	Unmapped Scaffold_8	**GA29087**	GJ13167	47C
**21**	CG1041	3R, (83E)	**GA28753**	GJ14957	47C
**22**	CG40006	2L-HET	**GA28752**	GJ14946	47C
**23**	CG17018	2L-HET (40F7)	**GA14273**	GJ13178	47C
**24**	CG3057*	2L, (23A5)	**GA28751**		
**24**	CG3057*	2L, (23A5)	**GA28750**	GJ14937	47C

Both mitotic and polytene chromosome map of *D*. *melanogaster* heterochromatic genes are indicated.

The gene marked by the asterisk is intronless

As shown in Fig 5A and Table 3, 194 Kb of Dpse_63A harbors 17 genes; 13 are colinear with those located at Dvir_55D, 2 with those located at Dvir_53D, while 2 genes (numbered 15 and 16 in Table 3) are not present at either Dvir location. Together, 15 genes maintain the same transcription direction in both Dpse_63A and Dvir_55D-53D. This suggests that Dpse_63A results from a recombination event that occurred between Dvir_55D and Dvir_53D in an ancestral chromosome like that in *D*. *virilis*. Notably, the same gene arrangement expected to be produced by the reciprocal event of such a hypothetical recombination is found at Dpse_78B of *D*. *pseudoobscura*. This arrangement in turn matches perfectly (and hence is syntenic) with region Dmel_47C1-3 of *D*. *melanogaster* (Fig 5A, Table 4). Furthermore, 12 out 15 genes in these two syntenic blocks of the Muller C elements of *D*. *pseudoobscura* and *D*. *virilis* are found scattered on the Muller C element in *D*. *melanogaster* heterochromatin (Table 3 and Fig 4).

We compared the gene content of the Dpse_83A block of the Muller B element with that of the Dvir_47C syntenic block of the Muller B element (Fig 5B and Table 5). Within the 123 Kb region analyzed of Dpse_83A, 23 genes are found that are colinear with genes located within the 112 Kb of Dvir_47C, sharing same transcription direction. Among those genes, 13 are orthologs of *D*. *melanogaster* 2Lh genes (Muller B), whereas 5 are orthologs of *D*. *melanogaster* 2Rh genes (Muller C); two remaining genes are orthologs of still unmapped *D*. *melanogaster* heterochromatin genes (CG12423 and CG17878). Thus, 20 out of 23 genes in Dvir_47C and Dpse_83A syntenic blocks are embedded in pericentric heterochromatin in *D*. *melanogaster*.

As shown in Table 1, among the *D*. *melanogaster* 2Rh gene orthologs found, 11 map to Dpse_82AB and 18 map to Dvir_42F-43A. A detailed comparative analysis of Dvir_42F-43A and Dpse_82AB cannot be performed, because Dpse_82AB is not assembled in a continuous scaffold and the orthologs are either not annotated or found in different scaffolds, most of which are unmapped (Table 6). Thus, we used the assembled *D*. *virilis* genome sequence to further analyze 400 Kb of the Dvir_42F-43A region. 50 genes (from CG42595 to CG5277) are located in this region, (Dvir:scaffold_12963:13,968,303..14,371,342; Table 6). As previously shown (Table 1), 18 genes of Dvir_42F-43A (Muller B) were confirmed to be orthologous to genes of *D*. *melanogaster* 2Rh (Muller C). Our FISH mapping has also shown that 11 of these genes map to Dpse_82AB (Muller B), while 5 of them were previously not annotated (Table 1). It is therefore conceivable that Dvir_42F-43A is syntenic with Dpse_82AB, both regions sharing clustered *D*. *melanogaster* 2Rh gene orthologs.

**Table 6:** List of *D*. *virilis* genes at Dvir_42F-43A orthologous to genes of *D*. *melanogaster* and *D*. *pseudoobscura*.

D_melanogaster		Dvir_42F-43A	D_pseudoobscura		
Name	Chr. position		Name	Chr. position	Notes
CG42595 (*l41ad*)	2R-HET	GJ22088	Not annotated	**82AB**	(this study) Yes in D_per
CG12547	2R-HET (41C2)	GJ17513	Not annotated	**83A**	(this study) Yes in D_per
CG41378	2R-HET	GJ22077	Not annotated	Not mapped	No in D_per
CG10424	3L, (75F7)	GJ22066	GA25285	4:group_1	Muller B
CG17691	2R-HET	GJ17514	GA29202	**82AB** U:group_17	(this study)
CG17510 (*Fis1*)	2R-HET (41F5)	GJ22057	GA14535	**82AB** U:group_493	(this study)
CG17665	2R-HET	GJ17515	Not annotated	**82AB** U:group_493	(this study)
-	-	GJ17516	-	-	Low score
CG17883	2R-HET (41B3)	GJ22046	GA25512	**82AB** U:group_456	(this study)
CG40498	2R-HET	GJ26084	Not annotated	**82AB** U:group_17	(this study)
CG40080(*Haspin*)	2R-HET	GJ22035	GA25640^**b**^	**82AB** U:group_17	(this study)
UnKnown		GJ17517	Not annotated	U:singleton_257	Unknown
CG45781	2R-HET	GJ22025	GA28727	**82AB** U:group_221	(this study)
UnKnown		GJ22003	GA24011	U:group_154	
UnKnown		GJ22014	Not annotated	Not mapped	Yes in D_per
UnKnown		GJ17156	UnKnown		Low score
CG40196 (*Maf1*)	2R-HET	GJ21993	Not annotated	4:group_2 1,189,031..1,188,032	Muller B
CG12559 (*rolled*)	2R-HET	GJ17518	Not annotated	**82AB** U:group_48 U:group_246	(this study)
-	-	GJ25980	-	-	-
CG41265 (*l41ab*)	2R-HET	GJ21982	Not annotated	**94A** 4:group_3	(this study)
CG17684	2R-HET	GJ21972	GA25532	**93D** 4:group_2 1190918..1200457 (-)	(this study)
CG5603 (*CYLD*)	2L, (31C7-D1)	GJ17519*	GA27711	4:group_2 1221653..1224709 (-)	Muller B
CG5604	2L, (31C4-6)	GJ21961*	Not annotated	U:singleton_791	Muller B
CG5362 (*mdh-1)*	2L, (31E1)	GJ17520*	Not annotated	Not mapped	Yes in D_per
CG5056	2L, (31D10)	GJ17521*	GA27750	4:group_2 1227267..1229151 (+)	Muller B
CG4968	2L, (31D1)	GJ21950*	GA18560	U:group_92	Muller B
CG13138	2L, (31D1)	GJ21939*	GA27630	U:singleton_791	Muller B
CG18144 *(Hand)*	2L, (31C6-7)	GJ17522*	GA14815	U:group_3	Muller B
CG5384 *(Usp14)*	2L, (31D4-5)	GJ17524*	GA18840	4:group_2 1231213..1233560 (-)	Muller B
CG18619	2L, (31B1)	GJ17525*	GA15016	4:group_1	Muller B
CG5385	2L, (31D5-8)	GJ17526*	Not annotated	Not mapped	Yes in D_per
CG5655	2L, (31B1)	GJ17527*	GA19037	U:group_3	Muller B
CG5671	2L, (31B1)	GJ17528*	GA19047	U:group_3	Muller B
CG5640 (*Utx*)	2L, (31C7)	GJ21917*	GA27749	4:group_2 1203345..1219792 (+)	Muller B
CG5381	2L, (31D6-7)	GJ21906*	GA18837	U:group_3	Muller B
-	-	GJ26652	-	-	-
CG2944 (*gus*)	2R-HET (41D3)	GJ21895	GA23998	**82AB**U:group_200	(this study)
CG5676	2L, (31B1)	GJ17529*	GA19050	U:group_3	Muller B
CG9935	4, (102D1)	**GJ17530**	GA22545	U:group_233	pseudogene
CG15848 (*Scp1*)	2R-HET	Within GJ25984	Not annotated	**82AB** U:singleton_2543 U:group_418 U:group_462	(this study)
CG5680 (*bsk*)	2L, (31B1)	GJ21884*	Not annotated	Not mapped	Yes in D_per
CG13140 (*dpr19*)	2L, (31D11)	GJ21873*	GA12074	4:group_2 1111976..1119527 (+)	Muller B
CG17704 *(nippedB)*	2R-HET (41B3-C1)	**GJ25920**	GA27714	4:group_2 1123715..>1127389 (-)	Muller B
CG4916 (*me31B*)	2L, (31B1)	GJ17531*	Not annotated	U:group_796 (partial)	Muller B Yes in D_per
CG42596	2R-HET	**GJ21862**	Not annotated	**93A** U:group_3	(this study)
CG5694	2L, (31B1)	GJ17532*	GA19063	U:group_132 (partial)	Muller B
CG5300	2L, (31E1)	GJ21851*	GA18793	4_group_1	Muller B
CG31715	2L, (31D9)	GJ17533*	GA22482	U:group_525	Muller B
CG5271	2L, (31E1)	GJ21841*	GA24218	U:singleton_3680	Muller B
CG5277	2L, (31E1)	GJ21832*	Not annotated	U:singleton_3680 (partial)	Muller B Yes in D_per

Orthologs of genes located in region 31 (2L) of *D*. *melanogaster* are marked by an asterisk

The analysis of the Dvir_42F-43A region also revealed an intriguing result. Adjacent to the cluster of Dmel_2Rh genes orthologs, we found another gene cluster which contains orthologs of genes located in polytene chromosome region 31 (Muller B) of *D*. *melanogaster* (Table 6). Interestingly, in *D*. *pseudoobscura* most of the orthologs are found in unmapped scaffold, that usually contains genomic regions derived from heterochromatic regions [34,36].

### The nature of Dpse_63A, Dpse_83A and Dpse_82AB regions of *D*. *pseudoobscura*

The Dpse_63A, Dpse_83A and Dpse_82AB blocks are proximal to chromocentric heterochromatin. Interestingly, hybridization signals produced by most gene probes mapping to Dpse_63A, Dpse_83A and Dpse_82AB tend to exhibit a dispersed and grainy morphology (Fig 3), compared to the sharp euchromatic signals. This morphology is distinctive of sequences derived from partially polytenized heterochromatin found in the so-called β-heterochromatin of the chromocenter [50,51]. Other peculiar characteristics of pericentromeric heterochromatin in *D*. *melanogaster* are the enrichment of repeated sequences and the increased size of gene introns, compared to euchromatin [52].

We then asked whether the repositioning of gene clusters in regions located proximal to the chromocenter in *D*. *pseudoobscura* (Fig 4) was accompanied by an increase repeated sequences and/or in intron size. To this aim, we investigated the repeat content using RepBase analysis (see Materials and Methods). The results are shown in S2 Fig. The block Dpse_63A contains 21.4% repeats, while only 0.2% is found in the syntenic block Dvir_55D and 6.0% in Dvir_53D. The block Dpse_83A contains 9.0% repeats, while 1.7% was observed in the syntenic Dvir_47C. A precise repeat content of block Dpse_82AB cannot be estimated because this region is found fragmented in several unmapped scaffolds (see Table 6) which are likely to correspond to repeat-rich heterochromatin regions [34,36]. The repeat content of the inferred syntenic block Dvir_42F-43A was estimated to be low, around 3.0%. Thus, it appears that the repositioning of gene clusters from *D*. *virilis* to *D*. *pseudoobscura* pericentric regions has occurred concurrently with an increase of repeats, which mainly correspond to transposable element-homologous sequences (S2 Fig).

The intron size estimates of assuredly homologous introns found in the coding regions of genes present in the syntenic blocks are reported in S2 Table. Intronless genes and genes with unknown intron size have been not considered; genes with partially sequenced introns have been included in the analysis, which may give rise to understimated intron size. Genes from the Dpse_78B/Dvir_53D-55D euchromatic gene cluster have been used as control.

This analysis revealed that intron size of genes in the syntenic clusters Dpse_63A/Dvir_55D and Dpse_83A/Dvir_47C remains stable, with a mean value ranging from 427 bp at Dpse_63A to 597 bp at Dvir_55D, and from 362 bp at Dpse_83A to 418 bp at Dvir_47C. An increase in intron size was observed in genes of the proximal syntenic blocks Dpse_82AB/Dvir_42F-43A; in this case the mean value of Dpse_82AB cluster is about 2 fold that of Dvir_42F-43A cluster and is mainly due to the intron increase of 2 orthologs (CG12559 and CG15848) among the 10 genes present in the clusters. A consistent intron size increase is indeed observed in the *D*. *melanogaster* orthologs of the three above mentioned clusters. The mean values being 10 fold higher for orthologs of Dpse_63A cluster and 7–8 fold higher for those of Dpse_83A and Dpse_82AB.

Taken together, it appears that the repositioning of the analysed gene clusters from *D*. *virilis* to *D*. *pseudoobscura* has occurred concurrently with an accumulation of repeats, while the increase in intron size has been limited. By contrast, a significant increase in intron size of *D*. *melanogaster* heterochromatic genes is apparent.

### Immunolocalization of HP1a on polytene chromosomes of *D*. *virilis* and *D*. *pseudoobscura*

**Fig 6:**
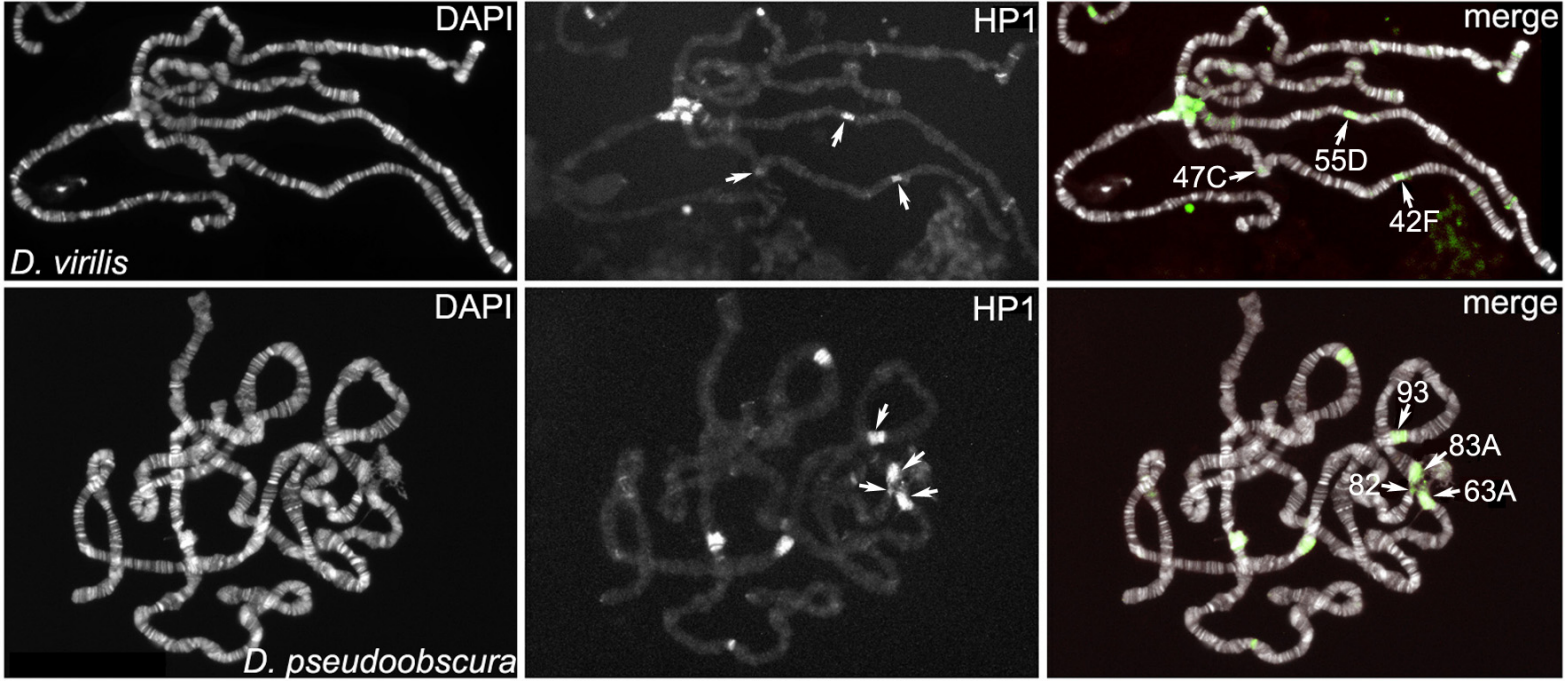
Immunolocalization of HP1a on polytene chromosomes. HP1a distribution is shown on polytene chromosomes of *D*. *virilis* (upper panels) and *D*. *pseudoobscura* (lower panels). The HP1a fluorescence signals clearly marks Dvir_42F-43A, Dvir_47C and Dvir_55D and to Dpse_63A, Dpse_83A, Dpse_82AB and Dpse_93. Three additional strongly stained regions are present in *D*. *pseudoobscura*.

**Fig 7:**
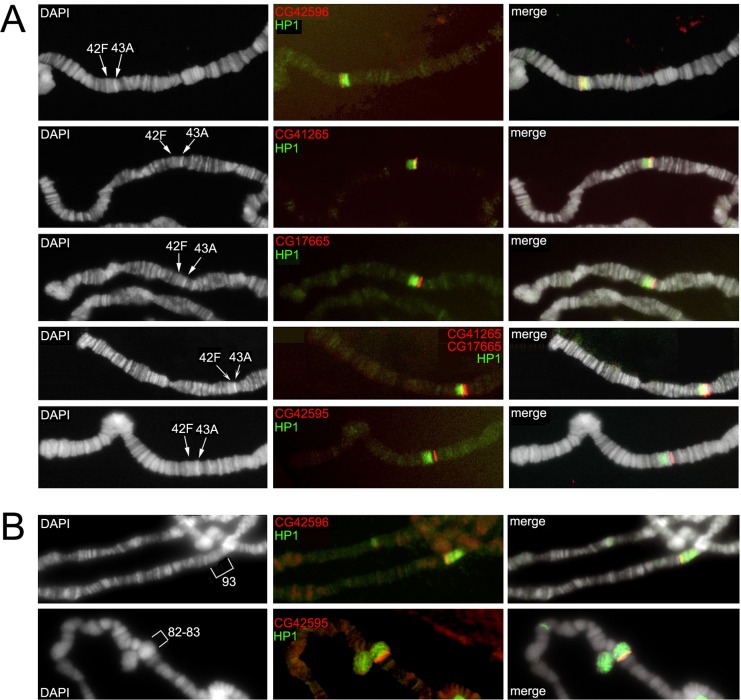
Sequential IF/FISH experiments performed on polytene chromosomes of *D*. *virilis* and *D*. *pseudoobscura* with anti-HP1a and specific gene probes. (A) In *D*. *virilis*, the FISH signals of CG41265 (GJ21982) and GJ21862 (CG42596), shown in red, map to the proximal and distal ends of region stained by anti-HP1a antibody (green). The FISH signals of CG42595 (GJ22088) and CG17665 (GJ17515) map to Dvir_43A. (B) In *D*. *pseudoobscura* the FISH signal of CG42595 ortholog is included in the large HP1 signal mapped to Dpse_83A; the FISH signal of CG42596 ortholog is included in the distal portion of the HP1 signal mapped to Dpse_93A.

The genomic organization of region Dvir_42F-43A in *D*. *virilis* opens an interesting issue. Although the polytene region 31 lies in the euchromatin of *D*. *melanogaster*, it shows some heterochromatic features; it has a poorly visible banding pattern with a “gooseneck” chromosomal morphology and shows abundant association with the heterochromatin protein HP1a, an evolutionarily conserved epigenetic mark very abundant in heterochromatin [53,54,55]. To test whether this association was evolutionarily conserved, we performed immunofluorescence (IF) experiments on *D*. *virilis and D*. *pseudoobscura* polytene chromosomes using anti-HP1a antibodies. The results clearly show that HP1a is present at Dvir_42F-43A (Fig 6) and remarkably also at Dvir_47C and Dvir_55D, where the other gene clusters are located. Similarly, in *D*. *pseudoobscura* HP1a is present at polytene regions Dpse_63A, Dpse_82AB and Dpse_83A where it colocalizes with the gene clusters (Fig 6). To further investigate the association of HP1a with the gene clusters, we performed sequential IF/FISH experiments where IF with anti-HP1a antibodies was followed by FISH using specific probes. We focused our attention on the Dvir_42F-43A cluster, using PCR probes of the GJ22088, GJ17515, GJ21982 and GJ21862 genes which are orthologous to the CG42595, CG17665, CG41265 and CG42596 heterochromatic genes of *D*. *melanogaster*, respectively (Table 6). As shown in Fig 7A, the FISH signals of CG41265 (GJ21982) and CG42596 (GJ21862) clearly colocalize with the strong HP1a signal found at division Dvir_42F. In particular, CG41265 (GJ21982) and CG42596 (GJ21862) map to the proximal and distal end of the HP1 region, respectively. The FISH signals of CG42595 (GJ22088) and CG17665 (GJ17515), on the other hand, map to Dvir_43A and therefore are linked to the HP1a signal, but not included in it. Interestingly, in *D*. *pseudoobscura* the CG42595 ortholog is included in the large HP1 signal mapped to Dpse_83A, while the CG42596 ortholog maps to Dpse_93A, a region located in the middle of euchromatin, which is also strongly stained by the anti-HP1 antibody (Fig 7B). Together, these results reveal an unexpected, evolutionarily conserved association between HP1a and the clusters of heterochromatic gene orthologs, even though not all genes in the cluster are included in the region decorated by the anti-HP1a antibody.

## Discussion

To investigate the evolutionary dynamics of heterochromatin, we have studied 53 heterochromatic genes of *D*. *melanogaster* chromosome 2, asking whether their current location in the genome originated from random events or arose as a consequence of specific rearrangements occurring during chromosome evolution. Among the genes, 17 map to the mitotic heterochromatin region h35, while 36 are spread out along a large portion of 2R mitotic heterochromatin spanning bands h41-h46 (Fig 1) [41,52].

The results of this analysis provide evidence that the orthologs of current *D*. *melanogaster* heterochromatic genes are clustered at three main syntenic regions in *D*. *virilis* and *D*. *pseudoobscura* (Table 1, Fig 4 and Fig 5). Interestingly, in *D*. *virilis* the genes are present across a few hundred kb, while in *D*. *melanogaster* the genes are found scattered over several Mb of DNA. Moreover, in *D*. *virilis* the gene clusters are located in the middle of euchromatin, in regions with low repeat content, while in *D*. *pseudoobscura* the clusters are associated with the distal portions of pericentric heterochromatin and are enriched in repeated sequences. Notably, the accumulation of repeated sequences in pericentric heterochromatin during Drosophila evolution it is a well known phenomenon [56] which is unlikely to be reversible.

Based on the Drosophila phylogeny (Fig 2; and ref 57,58) and on trasposable element enrichment of *D*. *pseudoobscura* clusters compared to those of *D*. *virilis* (S2 Fig), we propose that the ancestral clusters were located in euchromatin and underwent repositioning into pericentromeric regions in the lineage that gave rise to the *melanogaster* subgroup species [57,58]. A similar trend has been previously suggested for the evolution of a cluster of seven 2Lh genes of *D*. *melanogaster* (30). However, further analyses are needed to fully test this hypothesis.

Remarkably, we have also found that in both *D*. *virilis* and *D*. *pseudoobscura* the clusters show a striking association with the HP1a protein (Figs 6 and 7), one of the most highly evolutionarily conserved epigenetic marks of heterochromatin [59, 60, 61].

The only exception to the clustering is given by seven genes (CG17684 CG41265, CG42596, CG17678, CG17493, CG40285 and CG40129) that appear to have moved individually (Table 1 and Fig 4). In mammalian genome evolution, transposition events can drive the movement of individual genes from sex chromosomes to autosomes [62]. Similarly, in Drosophila phylogeny, movements of individual genes have occurred by retrotransposition and DNA-based transposition [63]. The mechanism of movement of these seven genes is unlikely to be due to retrotransposition or recombination, as suggested by their conserved exon-intron structure (S3 Fig), In fact, CG17684 CG41265, CG42596, CG17678 show an identical intron-exon structure in the three species. CG17493 is intronless in all the species, CG40285 is intronless in *D*. *virilis* and *D*. *pseudoobscura*, but carries a single intron in *D*. *melanogaster*, while CG40129 has a similar complex intron structure in both *D*. *melanogaster* and *D*. *virilis* and it is not assembled in *D*. *pseudoobscura*. It is then possible that the above mentioned seven genes may have moved by DNA-based transposition, although the molecular basis underlying their chromosomal evolution needs further investigations.

### The genes at the pericentric regions of Muller C and Muller B elements

In the Muller C element of *D*. *pseudoobscura* we found a cluster of 13 orthologs of *D*. *melanogaster* 2R heterochromatin, located at ± 200 Kb DNA from the proximal region Dpse_63A. In the Muller C element of *D*. *virilis*, 10 out of these 13 orthologs map to the euchromatic regions Dvir_55D, 2 map to Dvir_53D, while one (CG30438) was not detected in these regions.

Our analysis suggests that the arrangement of genes within Dpse_63A arose by recombination between Dvir_55D and Dvir_53D in an ancestral Muller C element like that of *D*. *virilis* (Fig 5A). Evidence for this recombination event relies on the proximity of GJ20406 and GJ22228 at Dpse_63A and reciprocally by the proximity of GJ20124 to GJ21920 found at Dpse_78B (Tables 3 and 4). The junction GJ20406-GJ22228 is present in the obscura group species, in *D*. *ananassae* and in *D*. *willistoni*, but not in the melanogaster subgroup species (*D*. *erecta*, *D*. *yakuba*, *D*. *sechellia*, *D*. *simulans*, *D*. *melanogaster*), whereas the junction GJ20124-GJ21920, representing the reciprocal event, is found in all the species analyzed except *D*. *willistoni*. Moreover, the GJ20406-GJ22228 association is almost proximal to the pericentromeric heterochromatin as seen in *D*. *pseudoobscura*, while the GJ20124-GJ21920 association maps to the euchromatin in all the analyzed species.

Taken together, these observations suggest that: i) the hypothetical recombination event occurred in the ancestor of the lineage that led to the subgenera Drosophila and Sophophora and ii) the two reciprocal events have had different evolutionary fates. Indeed, in the melanogaster subgroup species, gene order was lost in the pericentromeric region, yet was maintained in euchromatic regions. Furthermore, when we compare the gene order in the syntenic blocks Dpse_63A/Dvir_55D-53D with that found in the *D*. *melanogaster* 2R heterochromatin (Table 3), a scrambled arrangement of the 13 orthologous heterochromatin genes is apparent, suggesting that recurrent internal rearrangements occurred during chromosome evolution of this pericentric region.

Dpse_83A and Dpse_82AB adjacent regions are located proximally to the chromocenter in the *D*. *pseudoobscura* Muller B element and harbor at least 29 orthologs of *D*. *melanogaster* heterochromatic genes. Dpse_83A contains 13 orthologs of Dmel_2Lh and at least 5 orthologs of Dmel_2Rh (Fig 5B, Table 5), while the Dpse_82AB carries 11 orthologs of Dmel_2Rh (Table 1). Our data indicate that Dpse_83A and Dpse_82AB are syntenic with Dvir_47C and Dvir_42F-43A of the Muller B chromosome (Tables 5 and 6). The novelty of this observation is that during chromosome evolution, further rearrangement must have occurred to separate the two contiguous regions Dpse_82AB and Dpse_83A, giving rise to the relocation of a group of genes from these two Muller B regions to a pericentromeric Muller C region in the descendent lineage, that is the *D*. *melanogaster* 2R heterochromatin (Fig 4).

The mechanistic details responsible for this event are unknown, because extensive sequence data are not available for the Dpse_82AB-Dpse_83A regions in the *D*. *pseudoobscura* genome assembly. Some observations can, however, help to reconstruct the molecular events which occurred at the time of the fusion of two separated Muller B and Muller C arms into a single chromosome.

The first observation is the finding of duplicated sequences (CG3057) at Dpse_83A (Fig 5B and Table 5). Although these duplications cannot be associated with specific breakpoints, they might have arisen by staggered single-strand breaks, as postulated in the isochromatid model by Ranz et al. [64]. The second observation is the scrambled arrangement of the *D*. *melanogaster* heterochromatic genes compared to the gene order seen in the syntenic blocks Dpse_83A/Dvir_47C (Table 5) and in Dvir_42F-43A (Table 6), which would suggest numerous internal rearrangements during chromosome evolution. In addition, these rearrangements may have contributed to the expansion of DNA sequences, since the location of Dmel_2Rh genes spans megabases of DNA (from mitotic bands h41 to h46).

Notably, in Dvir_42F-43A the cluster of Dmel_2Rh gene orthologs is flanked by a cluster of *D*. *melanogaster* gene orthologs mapping to polytene region 31, in the middle of euchromatin (Table 6), a location known to be enriched in HP1a protein. We indeed found that the HP1a protein is associated with the gene cluster at Dvir_42F-43A (Fig 6) and also with the other clusters of both *D*. *virilis* and *D*. *pseudoobscura* (Fig 7). If such association represents an ancestral condition, then the *D*. *virilis* clusters may represent proto-heterochromatic sites whose repositioning and expansion led to the origin of pericentric regions of *D*. *melanogaster* chromosome 2.

Together, our results are consistent with a scenario where the *D*. *melanogaster* heterochromatin genes of chromosome 2 arose through an evolutionary repositioning from a euchromatic location (*D*. *virilis*) to heterochromatin, passing through an intermediate location in regions associated with distal heterochromatin (*D*. *pseudoobscura*). A similar trend was described by a study on the evolution of the heterochromatic gene cluster containing the *light* gene [30]. Recently, we have reported that the heterochromatic *Yeti* gene underwent a similar repositioning [65]. Here we showed that *Yeti* was part of a euchromatic gene cluster that was relocated by recombination to the heterochromatin of chromosome 2 (Fig 5A).

### HP1 and repositioning of gene clusters to pericentric heterochromatin

Previous studies have investigated the epigenetic states that enable the expression of heterochromatic genes and render their promoters heterochromatin-dependent [18,33], yet little is known about the evolutionary process(es) through which this has occurred. The results of the present study indicate that, in the *Drosophilidae* evolution, movement of genes from euchromatin to heterochromatin preferentially occurred by evolutionary repositioning of gene clusters which show close association with the HP1a protein (Figs 6 and 7**).** The molecular mechanisms responsible for repositioning of the large clusters are presently unknown, because of our poor knowledge of the breakpoints involved in the rearrangements and arrangements of genes flanking the regions under analysis. However, comparative studies carried out in Drosophila species have shown that during chromosomal evolution, transposable elements and other repetitive sequences have been implicated in the generation of rearrangements [34, 35, 40, 42, 64].

The association of *D*. *virilis and D*. *pseudoobscura* gene clusters with HP1a is indeed intriguing. Spellman and Rubin [66] have suggested that block conservation is needed to regulate a coordinated expression of the genes present in a block. There is evidence showing that HP1a, among its versatile functions as an element of the chromatin architecture and in gene silencing, also has a stimulating effect on the expression of both heterochromatic genes and region 31 genes [67, 68, 69].

Although not all genes within the clusters cytologically map to the HP1a-enriched portion (Fig 7), it is unlikely that such association arose by chance. It is worth noting that the association with HP1a is conserved even by genes that have moved away from the cluster (see the example of the Dmel_CG42596 ortholog found at Dpse_93A (Fig 7B). In addition, while the ortholog of Dmel_CG42595 lies at the very proximity of the HP1a signal at Dvir_43A, but is not included in it, in *D*. *pseudoobscura* it has become included in the HP1 signal at Dpse_83A.

Could we interpret this association with HP1a (and possibly with other epigenetic regulators) in an evolutionary perspective? If so, might the presence of HP1 in the ancestral domains have contributed to the success of gene repositioning into pericentric heterochromatin? Although the results of the present work cannot provide a final answer to these questions, some speculations about possible scenarios are possible.

We could envisage an evolutionary scenario where ancestral HP1-like association may have favored the expression of relocated gene clusters. It is well known that euchromatic genes moved into pericentric heterochromatin are subjected to position effect variegation (PEV) [21]. However, heterochromatic genes of *D*. *melanogaster* such as *light* and *rolled* are bound by HP1a which positively contributes to their expression [25, 26, 70]. We can speculate that genes in ancestral "proto-heterochromatic" clusters were already used to being active in an HP1a environment, and were hence less likely to exhibit deleterious position effects when moved to a pericentric region. Thus, at least in certain instances, the ancestral association with HP1 would have acted as an "epigenetic shield", protecting the relocated gene clusters against silencig effect, and favoring their expression in pericentromeric regions.

In addition, ancestral HP1-like may have contributed to the formation of rearrangements that eventually led to the gene cluster repositioning. For instance, HP1 may have mediated long-distance interactions between different HP1-chromatin domains (i.e., located in euchromatin and in pericentric heterochromatin). If DNA breaks occurred in physically close domains, then rearrangements may eventually be generated following end-joining repair events, thus promoting the repositioning of HP1-associated chromatin domains.

Although further experiments will be required to investigate the evolutionary significance of the association of HP1 with the *D*. *virilis* and *D*. *pseudoobscura* gene clusters, our work provides previously unanticipated results that will contribute to the understanding of the molecular evolution of genes embedded in pericentric heterochromatin.

## Materials and Methods

### Drosophila species and gene nomenclature

The following species were used for the cytological preparations: *D*. *pseudoobscura* (14011–0121.94), and *D*. *virilis* (15010–1051.00), from Tucson Drosophila Stock Center, University of Arizona, USA. Polytene chromosomes were prepared according to Pardue [71]. Gene names reflect the species-specific nomenclature, or use the *D*. *mel* gene symbol.

### Molecular probes

Species-specific probes were generated by PCR on genomic DNAs. Opposite primers were selected on the basis of published sequenced genomes and chosen to avoid the inclusion of intronic sequences. The list and the size of the probes generated are in S1 Table. Primers obtained from the *D*. *persimilis* sequenced genome were used to amplify the orthologs CG12559, CG12547, CG42595, and CG41265 with *D*. *persimilis* (14011–0111.01) and/or *D*. *pseudoobscura* DNAs. Amplified DNA fragments were eluted from agarose gels and cloned in pGEM-T vector (Promega). In every case the plasmid probes were verified by DNA sequencing.

### Fluorescence in situ hybridization (FISH)

Fluorescence in situ hybridization (FISH) was performed according to Pimpinelli et al. [72].

Squashed preparations of polytene chromosomes from salivary glands dissected from third instar larvae of *D*. *virilis* were denatured and hybridized with Cy3-dCTP or FluorX-dCTP (GE Healthcare) labeled probes. CG41265 and CG17665 were simultaneously localized using mixed probes. Polytene chromosomes were stained with DAPI, 4’, 6’-diamidine-2’-phenylindole-dihydrochloride. Chromosome preparations were analyzed using a computer controlled Nikon E1000 epifluorescence microscope equipped with a cooled CCD camera (Coolsnap). Digital images were obtained using an Olympus epifluorescence microscope equipped with a cooled CCD camera. Gray scale images, obtained by separately recording Cy3, FluorX and DAPI fluorescence with specific filters, were pseudo colored and merged for the final image using Adobe Photoshop. Labelled sites were identified on the basis of published polytene maps [35].

### Immunofluorescence

Polytene chromosomes of *D*. *virilis* and *D*. *pseudoobscura* were HP1 immunostained according to James et al. [53] using the C1A9 anti-HP1 antibody. In brief, salivary glands were rapidly dissected in Cohen and Gotchell medium G containing 0.5% Nonidet P-40 and incubated in 2% formaldehyde fixative solution for 25 min. The preparations were incubated with monoclonal anti-HP1 C1A9 antibody (1:50) overnight at 4°C in a humid chamber. The slides were washed in TBS/0.05% Tween 20 three times for 5 min and incubated with secondary antibody 1:40 dilution of FluoroLink Cy2-labeled goat anti–mouse (Amersham Biosciences) for 1 h at room temperature in a humid chamber. Finally, the slides were washed three times in TBST at 4°C, stained with 4,6-diamidino-2-phenylindole (DAPI) at 0.01 μg/ml, and mounted in antifading medium.

### In silico search

Genomic Databases on the Flybase website (http://flybase.org; version FB2014_06) were searched by TBLASTN using amino acids from individual translated exons of *D*. *melanogaster* heterochromatic genes. Only those genes lacking a clear orthologous member in the OrthoDB database were searched. To retrieve orthologs we did a tblastn search using the amino acids of single or of two contiguous exons of *D*. *melanogaster* over the genomes of *D*. *virilis* and *D*. *pseudoobscura* (or *D*. *persimilis* when the query produced partial information in *D*. *pseudoobscura*). When the results showed high scores (E-value < e-80) and most importantly, all the subject results were in the same plus or minus frame in adjacent sequences, we then assembled a complete coding region that was compared with the *D*. *melanogaster* gene structure. Splice junctions were analysed by visual inspection comparing exons in the pairwise alignment of two DNA sequences. By these criteria we were able to reconstruct a *bona fide* structure of the gene. Multiple sequence alignments were performed with ClustalW procedure available at EMBL-EBI (http://www.ebi.ac.uk) or with the multialin interface at http://multialin.toulouse.inra.fr. Assembly of exon-intron was obtained by manual inspection of high-scoring alignments obtained using the Blast tools with translated ORF of *D*. *melanogaster* proteins and supported by EST annotated sequences. The changes proposed to the orthologous heterochromatin genes for the bestfit alignments are reported in S3 Table.

To estimate the repeat content within syntenic blocks, DNA sequences were retrieved from Flybase and repeats were identified using Censor [73], implemented at Repbase (www.girinst.org/censor/index.php) and the Arthropoda dataset. The masked sequences correspond to transposable elements and no satellite/simple repeats were masked in the final output. The masked sequences were filtered out retaining only alignments longer than 100 bp and with similarity greater than 80%, roughly corresponding to a score greater than 800. These values were used as threshold in our analysis which allowed elimination of spurious matches that might inflate the repeat content and prevented as much as possible false positive cross-species matches. Bed files containing the masked positions were uploaded and visualized as custom tracks in Gbrowse (www.flybase.org).

## Supporting Information

S1 Fig Gene structure. Gene structure comparison among orthologous *D*. *melanogaster* heterochromatic genes retrieved by TBLASTN analysis over *D*. *pseudoobscura*, *D*. *persimilis* and *D*. *virilis* genomic sequences. The list of the variations supporting the alignments is reported in S3 Table.
(TIF)

S2 Fig Repeats content within syntenic blocks. The repeats found by RepBase analysis are highlighted in yellow. **(A)** The syntenic blocks Dpse_63A/Dvir_55D-53D. Percent repeats: Dpse_63A = 21,4; Dvir_55D = 0,2—Dvir_53D = 6,0.The large arrow in the lower diagram show the position of the breakpoint between Dvir_55D and Dvir_53D regions. **(B)** The syntenic blocks Dpse_83A/Dvir_47C. Percent repeats: Dpse_83A = 9,0; Dvir_47C = 1,7. **(C)** The Dvir_42F-43A region; Percent repeats = 3,0. **(D)** The repeats contents within two unmapped scaffolds of *D*. *pseudoobscura* (Dpse_Ugroup493 and Dpse_Ugroup17) containing Dmel-Het genes. Percent repeats: Dpse_Ugroup493 = 13,8; Dpse_Ugroup17 = 21,7.
(TIF)

S3 Fig Gene structure of “wandered” genes. Exon-intron structure of genes present in non syntenic regions.
(TIF)

S1 Table Primers. List of the primer used for cloning species specific probes.
(DOCX)

S2 Table Intron size variations. This table lists the lenght of individual introns of orthologous genes within: **A)** The syntenic blocks Dpse_63A/Dvir_55D-53D; **B)** Dpse_83A/Dvir_47C/; **C**) The genes at the Dvir_42F-43A region compared to the unmapped genes in Dpse_82AB and to Dmel-HET genes; **D**) The control euchromatic blocks Dpse_78B/Dmel_47C1-3/Dvir_53-55D.
(DOCX)

S3 Table Revision annotations. Proposed revision of orthologous Het genes annotations.
(DOCX)
