# Supplementary figures and images for: Comparative Genomic Analyses Provide New Insights into the Evolutionary Dynamics of Heterochromatin in Drosophila
Source: PLoS Genet. 2016 Aug 11;12(8):e1006212. doi: 10.1371/journal.pgen.1006212 (PMC4981424; doi:10.1371/journal.pgen.1006212)

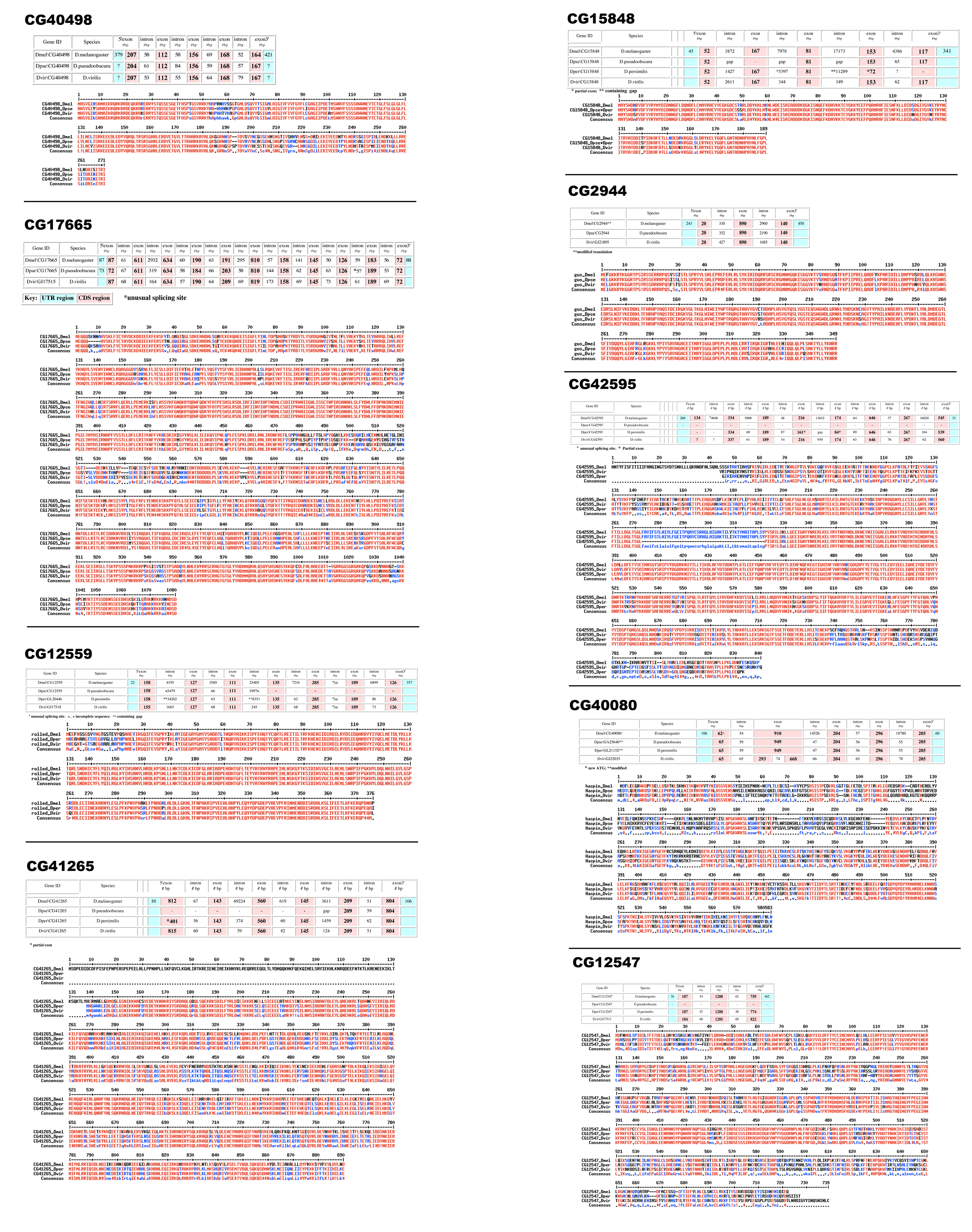

Supplement: S1 Fig — Gene structure comparison among orthologous D. melanogaster heterochromatic genes retrieved by TBLASTN analysis over D. pseudoobscura, D. persimilis and D. virilis genomic sequences. The list of the variations supporting the alignments is reported in S3 Table. (TIF) [file pgen.1006212.s001.tif]

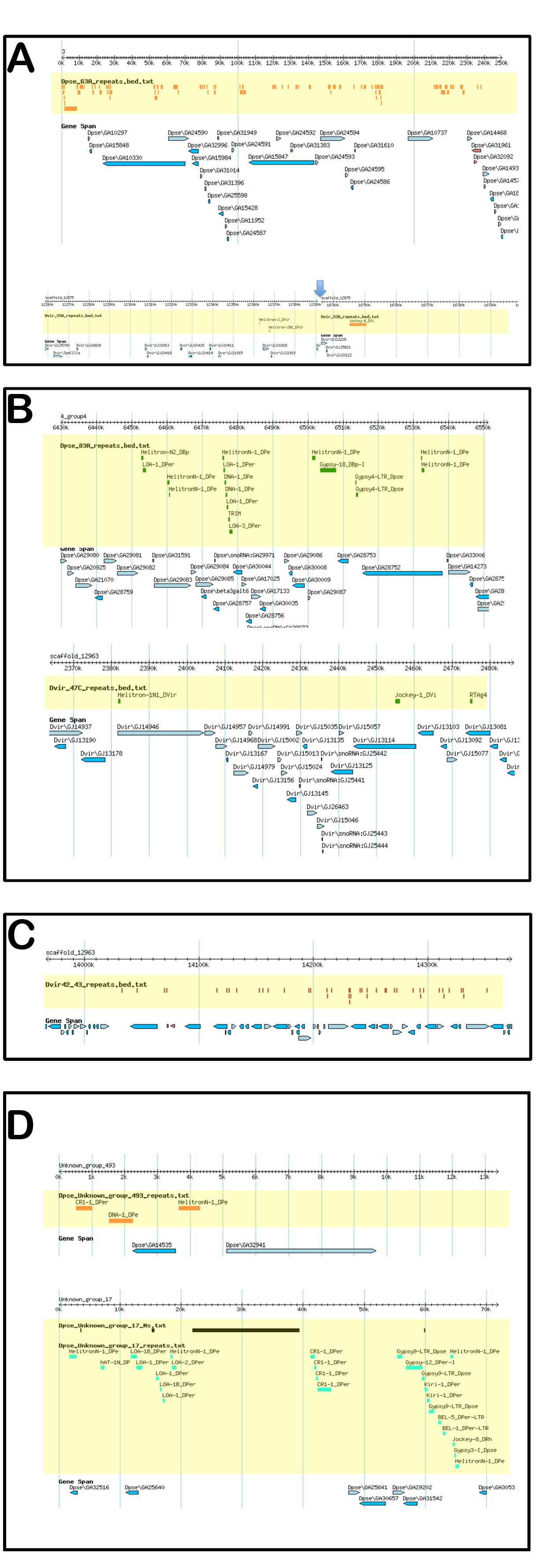

Supplement: S2 Fig — The repeats found by RepBase analysis are highlighted in yellow. (A) The syntenic blocks Dpse_63A/Dvir_55D-53D. Percent repeats: Dpse_63A = 21,4; Dvir_55D = 0,2—Dvir_53D = 6,0.The large arrow in the lower diagram show the position of the breakpoint between Dvir_55D and Dvir_53D regions. (B) The syntenic blocks Dpse_83A/Dvir_47C. Percent repeats: Dpse_83A = 9,0; Dvir_47C = 1,7. (C) The Dvir_42F-43A region; Percent repeats = 3,0. (D) The repeats contents within two unmapped scaffolds of D. pseudoobscura (Dpse_Ugroup493 and Dpse_Ugroup17) containing Dmel-Het genes. Percent repeats: Dpse_Ugroup493 = 13,8; Dpse_Ugroup17 = 21,7. (TIF) [file pgen.1006212.s002.tif]

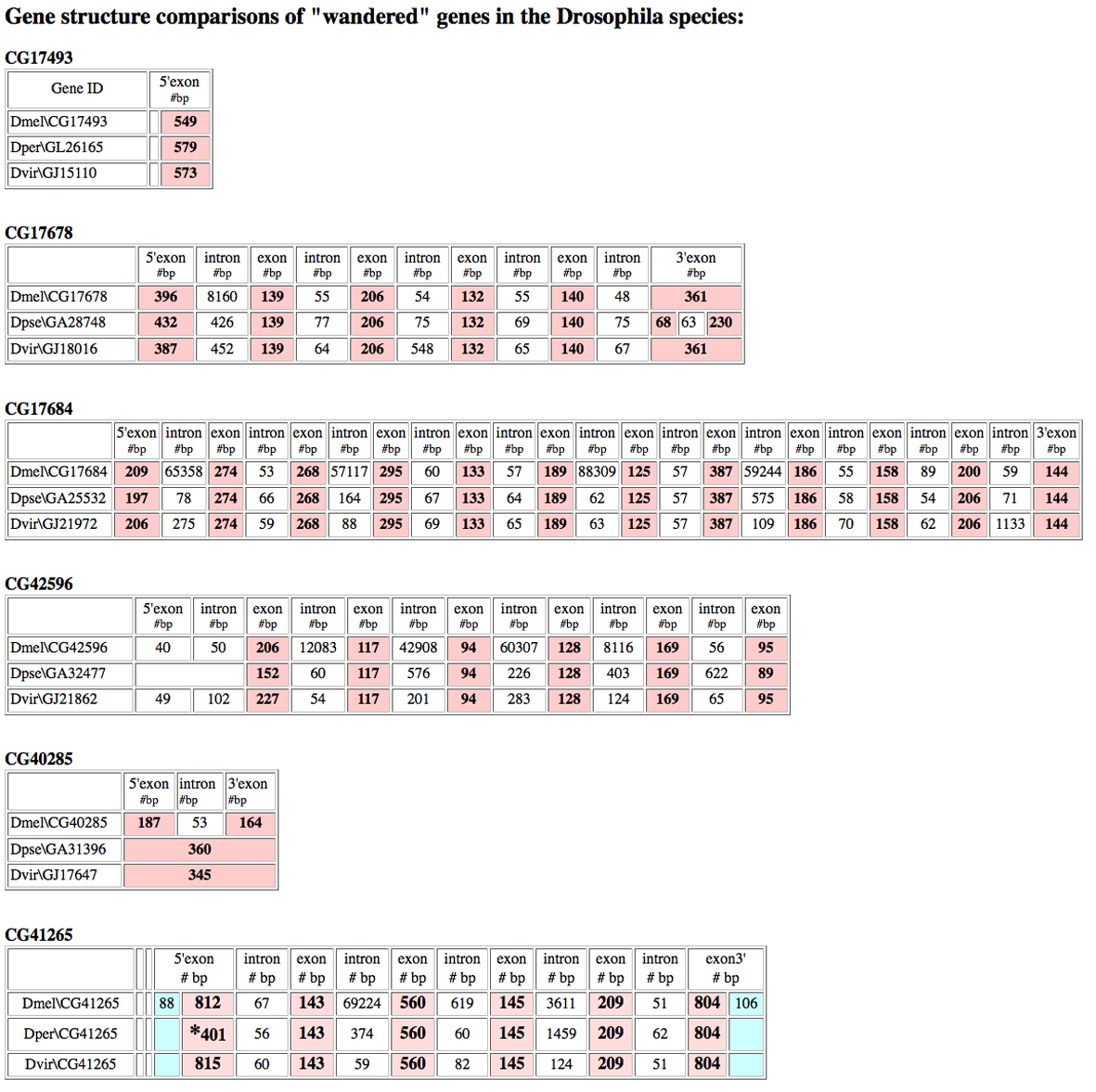

Supplement: S3 Fig — Exon-intron structure of genes present in non syntenic regions. (TIF) [file pgen.1006212.s003.tif]

**S1 Table.** List of the primers used for cloning species specific probes.


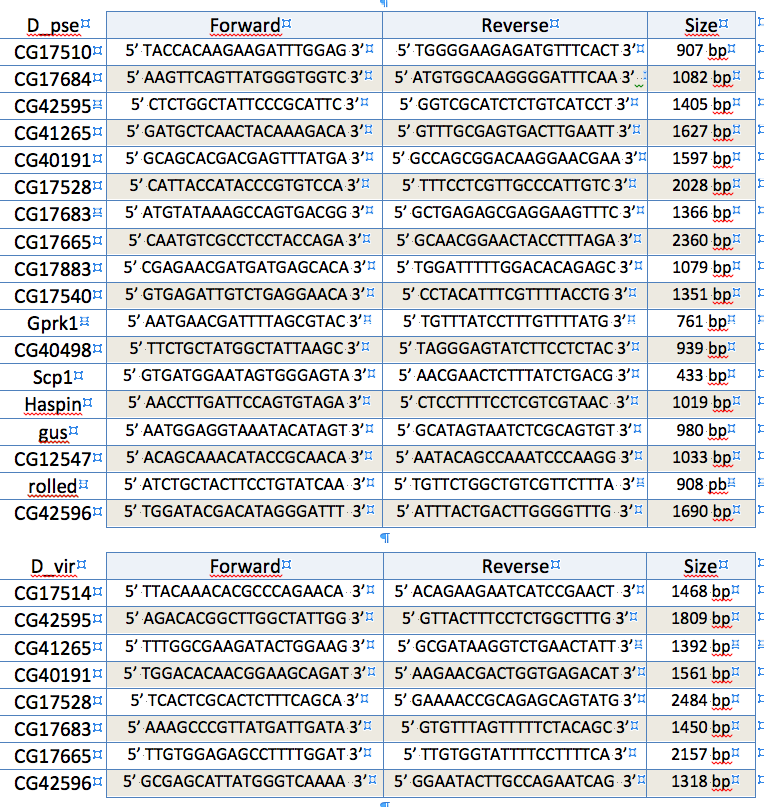

Supplement: S1 Table — List of the primer used for cloning species specific probes. (DOCX) [file pgen.1006212.s004.docx]
