# Supplementary material for: Comparative Genomic Analyses Provide New Insights into the Evolutionary Dynamics of Heterochromatin in Drosophila
Source: PLoS Genet. 2016 Aug 11;12(8):e1006212. doi: 10.1371/journal.pgen.1006212 (PMC4981424; doi:10.1371/journal.pgen.1006212)
Supplement: S2 Table — This table lists the lenght of individual introns of orthologous genes within: A) The syntenic blocks Dpse_63A/Dvir_55D-53D; B) Dpse_83A/Dvir_47C/; C) The genes at the Dvir_42F-43A region compared to the unmapped genes in Dpse_82AB and to Dmel-HET genes; D) The control euchromatic blocks Dpse_78B/Dmel_47C1-3/Dvir_53-55D. (DOCX) [file pgen.1006212.s005.docx]

**S2 Table.** Intron size variation among orthologs in the analysed species

| **A** | **Dpse 63A/Dvir 53D-55D** | **Dvir** | **Dpse** | **Dmel** |
| --- | --- | --- | --- | --- |
| **1** | CG10395 | 58 | 57 | 59 |
| **2** | CG30441 | 0 | 0 | 0 |
| **3** | CG10465 | 64 | 57 | 49 |
| **4** | CG40733 | 53 | 61 | - |
|  | (*RYa*) | 73 | 76 | - |
| **6** | CG3107 | 57 | 63 | 50 |
| **7** | CG33262 | 54 | 52 | 57 |
|  |  | 68 | 53 | 51 |
|  |  | 61 | 58 | 63 |
| **8** | CG2682 | 741 | 211 | 84 |
|  |  | 76 | 60 | 55 |
| **9** | CG1298 | 0 | 0 | 0 |
| **10** | CG3781 | 61 | 297 | - |
| **11** | CG40127 | 117 | 116 | 66 |
|  |  | 738 | 213 | 67 |
| **12** | CG33492 | 65 | - | 57 |
|  | (*Ir41A*) | 59 | 59 | 68 |
|  |  | 55 | 54 | 31597 |
|  |  | 52 | 59 | 25806 |
|  |  | 81 | 98 | 44451 |
|  |  | 55 | 52 | 59 |
|  |  | 57 | 59 | 54 |
| **14** | CG40218 | 69 | 57 | 69 |
|  | (*yeti*) |  |  |  |
| **17** | CG11066 | 5562 | 3204 | 2698 |
|  | (*scarface*) | 7045 | 5467 | 4286 |
|  |  | 71 | 70 | 55 |
|  |  | 67 | 63 | 62 |
|  |  | 73 | 59 | 1217 |
| **Mean value** |  | **597,384** | **427** | **4443,2** |
| St.Dev. |  | 1702,729 | 1222,373 | 11929,597 |

| **B** | **Dpse 83A/Dvir 47C** | **Dvir** | **Dpse** | **Dmel** |
| --- | --- | --- | --- | --- |
| **1** | CG17683 | 56 | 77 | - |
|  | (*l(2)41Ae*) | 53 | 59 | - |
|  |  | 59 | 73 | 57 |
| **2** | CG8245 | 69 | 52 | 60 |
|  |  | 88 | 56 | 61 |
| **3** | CG8426 | 112 | 167 | 65 |
|  |  | 60 | 170 | 79 |
|  |  | 307 | 64 | 58 |
|  |  | 650 | 65 | 70 |
|  |  | 80 | 55 | 61 |
|  |  | 66 | 59 | 59 |
|  |  | 721 | 189 | 2383 |
|  |  | 327 | - | - |
|  |  | 67 | 75 | 61 |
|  |  | 649 | 109 | 62 |
| **4** | CG1344 | 61 | 59 | 52 |
| **5** | CG17540 | 63 | 56 | 55 |
|  |  | 61 | 78 | 61 |
| **6** | CG17494 | 69 | - | - |
|  |  | 403 | 90 | 2988 |
|  |  | 57 | 65 | 59 |
| **7** | CG17528 | 89 | 63 | - |
|  |  | 216 | 74 | 58 |
|  |  | 114 | 76 | 65 |
|  |  | 64 | 76 | 63 |
|  |  | 60 | 73 | 54 |
| **8** | CG18028 | 56 | 53 | 56 |
|  | (*light*) | 56 | 68 | 52 |
|  |  | 69 | 61 | 50 |
|  |  | 462 | 55 | 67 |
|  |  | 60 | 60 | 1518 |
|  |  | 1837 | 1360 | 11008 |
|  |  | 58 | 238 | 61 |
|  |  | 63 | 50 | 48 |
|  |  | 59 | 78 | 54 |
|  |  | 57 | 61 | 52 |
|  |  | 66 | 69 | 59 |
| **9** | CG8734 | 0 | 0 | 0 |
| **10** | CG17489 | 179 | 172 | 158 |
|  | (*RpL5*) | 64 | 77 | 69 |
|  |  | 650 | 223 | 553 |
| **11** | CG17490 | 53 | 79 | 53 |
| **12** | CG3262 | 60 | 61 | 50 |
| **13** | CG1142 | 471 | 462 | 413 |
| **14** | CG3278 | 61 | 67 | 57 |
|  |  | 63 | 63 | 2340 |
|  |  | 58 | 63 | 60 |
|  |  | 65 | 61 | 3731 |
|  |  | 59 | 68 | 57 |
| **15** | CG12775 | 0 | 0 | 0 |
|  | (*RpL21*) |  |  |  |
| **16** | CG12567 | 2840 | 1683 | 11003 |
|  |  | 64 | 76 | 52 |
|  |  | 63 | 63 | 72 |
| **17** | CG40042 | 113 | 125 | 98 |
|  |  | 126 | 60 | 59 |
|  |  | 70 | 59 | 64 |
| **18** | CG40041 | 73 | 81 | - |
| **19** | CG12423 | 108 | 78 | 52 |
|  |  | 135 | 149 | 54 |
|  |  | 65 | 73 | 25795 |
|  |  | 64 | 81 | 60 |
| **21** | CG1041 | 86 | 87 | 79 |
|  |  | 51 | 67 | 65 |
|  |  | 61 | 64 | - |
|  |  | 74 | 61 | 63 |
|  |  | 66 | 63 | 59 |
|  |  |  |  |  |
| **22** | CG40006 | 7355 | 8472 | 4061 |
|  |  | 7796 | 6519 | 37374 |
|  |  | 224 | 271 | 156 |
|  |  | 87 | 65 | 54 |
|  |  | 1661 | 1565 | 29758 |
|  |  | 62 | 74 | 57 |
|  |  | 685 | 568 | 11915 |
|  |  | 972 | 1093 | 32041 |
|  |  | 67 | 92 | 56 |
|  |  | 333 | 441 | - |
| **23** | CG17018 | 65 | 63 | 898 |
|  |  | 195 | 129 | 52 |
|  |  | 61 | 74 | 62 |
|  |  | 87 | 58 | 68 |
|  |  | 75 | 63 | 69 |
| **Mean value** |  | **418,113** | **362,935** | **2553,422** |
| St.dev |  | 1239,562 | 1223,768 | 7511,877 |

|  |  |  |  |  |
| --- | --- | --- | --- | --- |
| **C** | **Dpse 82AB/Dvir 42F-43A** | **Dvir** | **Dpse** | **Dmel** |
| **1** | CG42595 | 6033 | ? | 4048 |
|  |  | 61 | 69 **^a^** | 5088 |
|  |  | 54 | 87 **^a^** | 48 |
|  |  | 930 | ? | 13652 |
|  |  | 63 | 89 **^a^** | 64 |
|  |  | 76 | 65 **^a^** | 57 |
|  |  | 62 | 104 **^a^** | 16820 |
| **2** | CG17691 | 133 | 69 | 573 |
|  |  | 60 | 67 | 51 |
|  |  | 67 | 51 | 59 |
| **3** | CG17665 | 68 | 67 | 61 |
|  |  | 164 | 319 | 2932 |
|  |  | 57 | 58 | 60 |
|  |  | 64 | 66 | 63 |
|  |  | 69 | 58 | 295 |
|  |  | 173 | 144 | 57 |
|  |  | 69 | 62 | 141 |
|  |  | 73 | 63 | 50 |
|  |  | 61 | 57 | 59 |
|  |  | 69 | 53 | 56 |
| **4** | CG17883 | 74 | 54 | 58 |
|  |  | 52 | 54 | 27 |
|  |  | 63 | 56 | 53 |
| **5** | CG40498 | 53 | 61 | 58 |
|  |  | 55 | 84 | 58 |
|  |  | 64 | 59 | 69 |
|  |  | 79 | 57 | 52 |
| **6** | CG40080 | 65 | 59 | 54 |
|  |  | 47 | 47 | 14526 |
|  |  | 56 | 56 | 57 |
|  |  | 55 | 55 | 18780 |
| **7** | CG45781 | 814 | 1471 | 6433 |
|  |  | 1224 | 842 | 56132 |
|  |  | 620 | 202 | 274 |
|  |  | 219 | 2071 | 51432 |
|  |  | 4322 | 3233 | 31503 |
|  |  | 691 | 60 | 52 |
|  |  | 1021 | 1107 | 62882 |
|  |  | 955 | 223 | 31210 |
| **8** | CG12559 | 1603 | >5479 * | 4195 |
|  |  | 68 | 66 | 3569 |
|  |  | 245 | >3997 * | 25205 |
|  |  | 68 | ? | 7216 |
|  |  | 54 | ? | 54 |
|  |  | 73 | ? | 6899 |
| **9** | CG2944 | 427 | 352 | 330 |
|  |  | 1685 | 2190 | 2960 |
| **10** | CG15848 | 2611 | 1427 * **^a^** | 3872 |
|  |  | 344 | >5397 * **^a^** | 7978 |
|  |  | 349 | >11289 * **^a^** | 17173 |
|  |  | 62 | 65 | 4386 |
| **Mean value** |  | **520,078** | **905,673** | **7878,058** |
| St.dev |  | 1096,186 | 2060,039 | 14726,996 |
|  | | | | |
| **D** | **Dpse 78B/Dvir 53D-55D** | **Dvir** | **Dpse** | **Dmel** |
| **1** | CG12340 | 57 | 61 | 61 |
|  |  | 60 | 59 | 578 |
|  |  | 1743 | 59 | 62 |
| **2** | CG12935 | 58 | 59 | 58 |
| **3** | CG7637 | 58 | 63 | 65 |
| **4** | CG12936 | 0 | 0 | 0 |
| **5** | CG12341 | 0 | 0 | 0 |
| **6** | CG7222 | 63 | 62 | 68 |
|  |  | 74 | 59 | 282 |
|  |  | 57 | 65 | 66 |
| **7** | CG12342 | 61 | 52 | 66 |
|  |  | 70 | 64 | 55 |
| **8** | CG12323 | 69 | 61 | 62 |
| **9** | CG12938 | 0 | 0 | 0 |
| **10** | CG12343 | 0 | 0 | 0 |
| **11** | CG12325 | 67 | 52 | 53 |
|  |  | 67 | 58 | 55 |
|  |  | 65 | 64 | 58 |
|  |  | 73 | 55 | 65 |
|  |  | 94 | 60 | 57 |
|  |  | 56 | 63 | 61 |
|  |  | 66 | 66 | 64 |
|  |  | 460 | 369 | 357 |
|  |  | 64 | 67 | 60 |
| **12** | CG12344 | 1773 | 556 | 606 |
|  |  | 2013 | 1072 | 1411 |
|  |  | 81 | 76 | 81 |
|  |  | 2543 | 1829 | 1417 |
|  |  | 75 | 84 | 58 |
|  |  | 73 | 60 | 115 |
|  |  | 84 | 73 | 90 |
|  |  | 159 | 67 | 98 |
| **13** | CG7686 | 57 | 67 | 59 |
| **Mean value** |  | **353,103** | **186,275** | **213,379** |
| St.dev |  | 692,514 | 379,567 | 363,244 |

**Notes**: Intronless genes have not been considered.

Genes with unknown intron size have been not considered.

Genes having partially sequenced introns are included in the analysis, giving rise to the understimated values.

* means containing intron gaps.

**a** means from *D. persimilis*.

The Dpse_78B/Dvir_53D-55D cluster represents a control euchromatic site.
