# Supplementary material for: Comparative Genomic Analyses Provide New Insights into the Evolutionary Dynamics of Heterochromatin in Drosophila
Source: PLoS Genet. 2016 Aug 11;12(8):e1006212. doi: 10.1371/journal.pgen.1006212 (PMC4981424; doi:10.1371/journal.pgen.1006212)
Supplement: S3 Table — Proposed revision of orthologous Het genes annotations. (DOCX) [file pgen.1006212.s006.docx]

**S3 Table.** Proposed revision of heterochromatic genes annotations.

| **Gene** | **FB annotation** | **Our analysis** | **Supported by** |
| --- | --- | --- | --- |
|  |  |  |  |
| **CG41265** | Not annotated | Dper_sc_1  1,480,916..1,485,248 | Multialign |
| **CG42595** (*uex*) | Not annotated | Dper_sc_16  262,364..267,523 (+) | Multialign |
| **CG40080** (*Haspin*) | GA25640 Dpse:U_group_17 10,931..12,994 (-) ATG start 12,568 | Dpse_U_group_17 10,999..12,936 (-) 5’exon different - ATG start 12,936 | Multialign |
| **CG12547** | GL21149 Dper_sc_16 250,917..256,140 [-] Stop at 253,680 | Dper_sc_16 256,140..253,859 [-] -3’exon different Stop at 253,859 | cDNA GenBank DR152705.1 |
| **CG2944** (*gus*) ATG at 2R:5,122,080 | GA23998 Dpse_U_group_200 14,240..19,049 [+] ATG start 14,479 | Dpse_U_group_200 15,041..18,070 [+] ATG start 15,041 | Multialign |
